# Shared properties of gene transfer agent and core genes revealed by comparative genomics of Alphaproteobacteria

**DOI:** 10.1099/mgen.0.000890

**Published:** 2022-11-09

**Authors:** Sonja Koppenhöfer, Jürgen Tomasch, Andrew S. Lang

**Affiliations:** ^1^​ Department of Biology, Memorial University of Newfoundland, St John’s, Newfoundland and Labrador, Canada; ^2^​ Laboratory of Anoxygenic Phototrophs, Institute of Microbiology of the Czech Academy of Science – Centre Algatech, Třeboň, Czech Republic

**Keywords:** codon usage, GC skew, genome plasticity, inversion, methylation, repetitive elements

## Abstract

Gene transfer agents (GTAs) are phage-like particles that transfer pieces of cellular genomic DNA to other cells. Homologues of the *Rhodobacter capsulatus* GTA (RcGTA) structural genes are widely distributed in the Alphaproteobacteria and particularly well conserved in the order Rhodobacterales. Possible reasons for their widespread conservation are still being discussed. It has been suggested that these alphaproteobacterial elements originate from a prophage that was present in an ancestral bacterium and subsequently evolved into a GTA that is now widely maintained in extant descendant lineages. Here, we analysed genomic properties that might relate to the conservation of these alphaproteobacterial GTAs. This revealed that the chromosomal locations of the GTA gene clusters are biased. They primarily occur on the leading strand of DNA replication, at large distances from long repetitive elements, and thus are in regions of lower plasticity, and in areas of extreme GC skew, which also accumulate core genes. These extreme GC skew regions arise from the preferential use of codons with an excess of G over C, a distinct phenomenon from the elevated GC content that has previously been found to be associated with GTA genes. The observed properties, along with their high level of conservation, show that GTA genes share multiple features with core genes in the examined lineages of the Alphaproteobacteria.

## Data Summary

Only publicly available genomic sequences, downloaded from the National Center for Biotechnology Information (NCBI), were used in this study. The accession numbers are provided in Table S1 (available in the online version of this article).

**Impact Statement**

Gene transfer agents (GTAs) are phage-derived genetic elements that mediate gene transfer in some prokaryotes. The genetic potential for GTA production is notably widespread in some lineages within the class Alphaproteobacteria. The functionality of most of these elements remains untested, but their conservation leads to speculation that they serve some beneficial function for these bacteria. We analysed the genomic properties of alphaproteobacterial GTA genes that might relate to their conservation. This revealed they have biased chromosomal locations such that they primarily occur on the leading strand of DNA replication, in regions of lower plasticity, and in areas of extreme GC skew. These are properties of core genes in these bacteria, providing further evidence of the importance of GTAs for the biology of these organisms.

## Introduction

Gene transfer agents (GTAs) are phage-like particles that transfer small pieces of genomic DNA between cells. They have been identified in multiple Gram-negative bacteria and one archaeon [1]. Currently there are five distinct GTA types known, each appearing to have an independent evolutionary origin and varying breadths of taxonomic distribution [1]. Homologues of the *Rhodobacter capsulatus* GTA (RcGTA) genes are found in the genomes of members of multiple orders of the class Alphaproteobacteria [2, 3], and the functionality of these RcGTA-like elements has been confirmed in divergent members of the alphaproteobacterial order Rhodobacterales [1, 4–6]. It has been suggested that these GTA elements are descendants of a prophage that integrated into the genome of an ancestral alphaproteobacterium, subsequently lost multiple phage-specific features, such as DNA replication and packaging specificity, and acquired mutations that resulted in a reduced head size [1]. This proto-GTA was then maintained through to the evolution of the extant lineages where the GTA genes are under cellular control.

Most of the RcGTA structural genes are located in a gene cluster of approximately 14 kb [7] that is conserved in the genomes of almost all examined members of the order Rhodobacterales [3]. This set of genes is also conserved to varying degrees in approximately half of the members of the alphaproteobacterial orders Rhizobiales, Sphingomonadales and Caulobacterales [3]. Possible reasons for the widespread conservation of these GTA genes are still being discussed. On the one hand, GTAs might contribute to the transfer of beneficial genes among cells [1, 7–9]. This hypothesis is supported by findings on the unrelated GTA produced by *Bartonella* spp., where particle release and DNA uptake are restricted to the subpopulations with the highest fitness [10] and thus the GTAs are more likely to transfer genes that offer a benefit to the recipient cell. However, a modelling approach did not find support for any fitness advantage to GTA-producing over non-GTA-producing populations, as the resulting gene transfer did not compensate for the loss caused by GTA release [11], which requires cell lysis of the producing subpopulation of cells [12, 13]. Perhaps GTAs are simply defective remnants of previously functional prophages [8], but this is difficult to reconcile with the findings that the RcGTA-related genes are under purifying selection [14] and that the production of RcGTA is co-regulated with the ability of cells to receive DNA from the particles [15]. Alternatively, an immunological function of GTAs has been proposed where GTAs transfer prophage DNA that can be incorporated into a recipient cell’s CRISPR/Cas array and thereby ‘vaccinate’ the cells before an actual infection takes place [11].

The RcGTA family gene clusters show an increased GC content [(G+C)/(A+T+G+C)] relative to the rest of the genome, which results from a bias in the encoded proteins to contain amino acids that have a lower carbon content [16]. This could be important for the production of GTAs during nutrient limitation, as observed for RcGTA [17]. A previous analysis of different factors associated with GTA gene expression [18] drew our attention to the localization of the GTA gene clusters in regions of especially high GC skew, which is the normalized ratio of guanine to cytosine [(G-C)/(G+C)] and different from absolute GC content, in two considered species, *R. capsulatus* and *Dinoroseobacter shibae *. Circular bacterial chromosomes can be divided into two halves, the right and left replichores, based on the orientation relative to the origin (*ori*) and terminus (*ter*) of replication. The GC skew typically has positive values on the right replichore and negative values on the left replichore as guanine and cytosine dominate on the leading and lagging strand, respectively [18–23]. This asymmetric distribution is thought to be largely driven by deamination of cytosine to thymine, which might be affected by DNA replication, since the distribution pattern matches replication directionality [23, 24]. This chromosomal composition bias is increased by some factors, such as an elevated growth rate [22], and decreased by others, such as recombination [25], and an overall more pronounced GC skew correlates with lower numbers of repeats [26]. Repetitive sequences and mobile genetic elements such as prophages can facilitate chromosomal rearrangements that reduce genomic stability, although these increase genomic plasticity and can provide an organism with greater adaptability [27–29].

Motivated by these previous observations related to DNA composition patterns, we performed a comprehensive genome sequence and structure analysis focused on patterns of GTA gene cluster conservation in four orders of the Alphaproteobacteria. This revealed trends in their localization, GC skew, codon usage and potential DNA methylation, and led to the overall conclusion that GTA genes share multiple properties with core genes in these bacteria.

## Methods

Analyses were carried out with R studio version 4.0.3 and relevant packages (Table 1).

**Table 1.** List of R (version 4.0.3) packages used in this study

Package name	Version	Reference
Tidyverse	1.3.0	
Biostrings	2.54.0	
GenomicRanges	1.38.0	
Ggbio	1.34.0	[59]
XML	3.99–0.3	
RCurl	1.98–1.1	
Ringo	1.50.0	[60]
BSgenome	1.54.0	
ggExtra	0.9	
DescTools	0.99.34	
coRdon	1.4.0	[61]
rlist	0.4.6.1	
genoPlotR	0.8.9	[60]
reorientateCircGenomes	0.0.1	This study

### Genome dataset and chromosome reorientations

Closed genomic sequences from bacteria within four alphaproteobacterial orders, the Rhodobacterales (*n*=147), Sphingomonadales (*n*=114), Caulobacterales (*n*=30) and Rhizobiales (*n*=462), were obtained from the National Center for Biotechnology Information (NCBI) GenBank assembly database (e.g. https://www.ncbi.nlm.nih.gov/assembly/?term=Rhodobacterales) on 12 March 2019. The accession numbers of the sequences used are provided in Table S1 (available in the online version of this article). We note that there have been subsequent taxonomic revisions among these bacteria but do not believe these detract from the utility or meaning of our analyses as based on this previous, long-standing taxonomic organization. The origin of replication (*ori*) was identified on each chromosome using Ori-Finder and default settings [30]. The ptt files were generated from gbff files (https://github.com/sgivan/gb2ptt#gb2ptt), downloaded from the NCBI database on 23 April 2019. Only chromosomes where one *ori* could be unambiguously identified were subsequently included in the investigation. We next determined the locations of the gene encoding the GTA major capsid protein (MCP) and found that all were located on the presumed major chromosomes (largest replicons) that were used for subsequent analyses.

Depending on the analysis, the positions of *ori* or the GTA MCP gene were used to reorient the DNA fasta and gff files using custom R functions that are available within the newly developed package ‘reorientateCircGenomes’ (https://github.com/SonjaElena/reorientateCircGenomes.git). This package simplifies reorientation of sequences within fasta and gff files that originate from the NCBI database or Prokka based on base pair location or ProteinID. It can also be used to visualize circular chromosomes with strand information, GC skew and locations of selected genes.

### Homology analysis

To identify homologous proteins, and thus gene families, in the genomes from the different orders, all proteins were blasted against each other and a matrix was generated for each order using Proteinortho version 5.16b [31]. The criteria to be considered a homologue were an e-value ≤1e-05, identity ≥30 % and coverage ≥75 %. Based on the identification of specific genes of interest (e.g. the GTA major capsid protein gene) in reference organisms’ genomes, this database was then used to identify homologues in the other genomes. The reference organisms for the Rhodobacterales, Sphingomonadales, Caulobacterales and Rhizobiales are *Dinoroseobacter shibae* DFL 12 (=DSM 16493)*, Sphingopyxis alaskensis* RB2256, *Brevundimonas subcrescentus* ATCC 15264 and *Brucella suis* 1330, respectively. A protein was designated a core protein if it was present in ≥90 % of all genomes within an order.

### DNA composition analysis

The GC skew was calculated as (G-C)/(G+C) for a sliding window of 10000 bp. For cumulative visualizations, the sliding window size was set to 0.1 % of the chromosome lengths and then the mean GC skew of all organisms being considered was calculated.

GC skew peaks were identified independently of the reversal of the right and left replichores by using sliding quantiles to identify the local GC skew minima and maxima. The positions on the chromosome with GC skew values that belonged to the upper or lower 3 % of the GC skew values in a sliding window of 150 000 bp were identified. Genes located at these locations were then identified for further analyses. The relative GC skew was calculated as (skew_sample_–skew_control_)/skew_control_. The GC content of genes was calculated as G+C over the length of each protein coding region.

To examine the GC skew of prophages in relation to their respective host genomes, the insertion positions on the chromosome were determined using PHASTER [32]. Hits that overlapped with GTA locations, identified by ProteinOrtho, were attributed to be part of GTAs. To compare the GC contents and skews of GTAs and phages, the genomic sequences of phages that infect bacteria in the four considered orders were downloaded from the NCBI virus database (on 4 November 2020). The GC contents and skews were determined over sliding windows of 1000 bp for the phages and per gene for the GTAs.

### Identification of repeats, methylation motif sites and large-scale inversions

Repetitive elements were identified with RepSeek [33]. Only repeats with a length >800 bp and identity >90 % were included to focus the analyses on long and highly similar repeats that are more prone to recombination [34]. We excluded overlapping repeats because these are probably not a major reason for large-scale chromosomal rearrangements. CcrM methylation potential was examined by searching for the GANTC motif in the DNA sequences. To rule out the possibility that the pattern we observed was caused by base composition instead of a possible methylation site, we also examined variations of this pattern that have equal base compositions (CGANT, CTGAN) (Fig. S1). Large-scale chromosome rearrangements were identified by visual inspection using MAUVE with the option progressiveMauve [35].

**Fig. 1.**
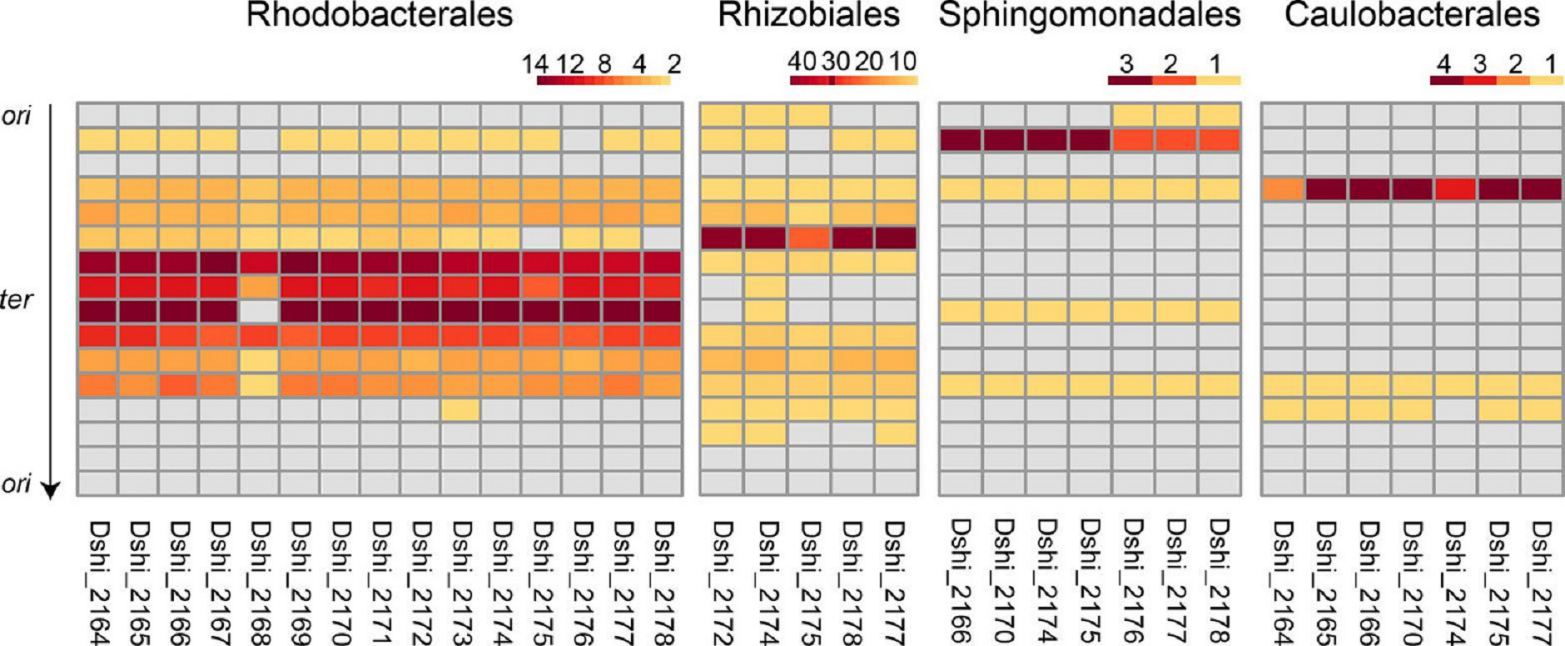
Localization of GTA gene cluster genes on alphaproteobacterial chromosomes. The chromosomes were normalized for scale, *ori*-oriented (top and bottom) and divided into 16 parts. Each gene is represented in one column and labelled according to the *D. shibae* locus tags (bottom); for reference, Dshi_2174 encodes the major capsid protein. The heatmap indicates the number of a gene’s homologues located in the region of the chromosome, according to the scales above, and grey indicates no homologue was found in a region. The differences in the numbers of individual genes detected in the orders is due to differences in the degrees of conservation of the gene cluster among the orders.

### Codon usage

The DNA sequences for each open reading frame (ORF) were extracted from the genome fasta nucleotide files, based on the position information in the annotation (gff) files using the Biostrings and Genomic Ranges packages in R (Table 1). After sorting the ORFs of each organism according to whether they were found in GC skew peaks or not, the occurrence of each codon in each group was counted. The means per codon for both groups and for each organism were then calculated. The relative codon usage was determined as (peak–not-peak)/not-peak. For visualization, codons were grouped based on the encoded amino acids.

### Phylogenetics

To identify closely related strains in which the location of the GTA gene cluster was switched between right and left replichores, a phylogenetic tree was generated for each order using RNA polymerase β protein (RpoB) amino acid sequences. Those RpoB sequences were identified with ProteinOrtho using sequences AAV96733.1, AAN30162.1, ANF54622.1 and ABF53199 as references for the Rhodobacterales, Rhizobiales, Caulobacterales and Sphingomonadales, respectively. The alignments and trees were generated with mega X [36]. The default settings were used for pairwise and multiple alignments. Partial deletion with a delay divergent cutoff of 30 % was used for gaps and missing data. The trees were constructed with the maximum-likelihood method. The branching patterns were evaluated using 100 bootstrap replicates, and the LG model was applied with gamma distribution at invariant sites. The site coverage cutoff was 95 %.

## Results and discussion

### Dataset generation

Closed genomic sequences were downloaded from the NCBI database for four orders of the class Alphaproteobacteria. These orders were chosen based on their high numbers of available complete genomic sequences and their high level of GTA gene cluster conservation [3]. The Rhizobiales had the most genomes (462), followed by the Rhodobacterales (147), Sphingomonadales (114) and Caulobacterales (30) (Table 2). To standardize analyses of gene localization, repeats and methylation motifs, all chromosomes were reoriented to the origin of replication (*ori*). Genomes were excluded when a single *ori* could not be unambiguously identified. For GTA gene cluster-related analyses, further dataset reduction was made based on the presence of the major capsid protein (MCP) gene. These selection criteria resulted in a reduced set of genomes available for analysis (Table 2). Repeating the analyses with only one representative strain per species for the Rhodobacterales and Rhizobiales showed that there was no bias in the results caused by overrepresentation of strains for certain species (data not shown).

**Table 2.** Number of genomes available for analysis based on selection criteria

Order	Closed genomes	One unambiguous origin of replication (*ori*)	GTA major capsid protein (MCP) gene^∗^	One representative strain per species
Rhizobiales	462	133	76	71
Rhodobacterales	147	70	59	45
Sphingomonadales	114	17	6	17
Caulobacterales	30	8	4	8

*Based on more than one representative strain per species; final number of genomes included in analysis.

### GTA gene clusters are located on the leading strand, close to the terminus of replication and far from repeat regions

As expected, based on previous analyses, the presence of complete GTA gene clusters was most well conserved in the genomes of Rhodobacterales members, followed by the Rhizobiales, Sphingomonadales and Caulobacterales (Fig. 1). The localization was conserved near the chromosomal replication terminus (*ter*) in the Rhodobacterales (Fig. 1) and to a lesser extent in the Rhizobiales. The clusters were more scattered in the Sphingomonadales and tended to be halfway between *ori* and *ter* in the Caulobacterales (Fig. 1).

Long repeats (>800 bp) enable homologous recombination [34] and can be responsible for extensive chromosomal rearrangements. Indeed, recombination events between repetitive elements were identified as the likely explanation for the GTA gene cluster’s location on different replichores in closely related species in the Rhodobacterales and Rhizobiales. For example, the replichore switch observed between *Phaeobacter inhibens* 2.10 and *P. galleciensis* was associated with regions containing many transposable elements (Fig. S2), which are often the cause of homologous recombination [37]. Similarly, the recombination event associated with replichore differences between *B. suis* and *Brucella abortus* was due to a region with paralogous genes (Fig. S2).

**Fig. 2.**
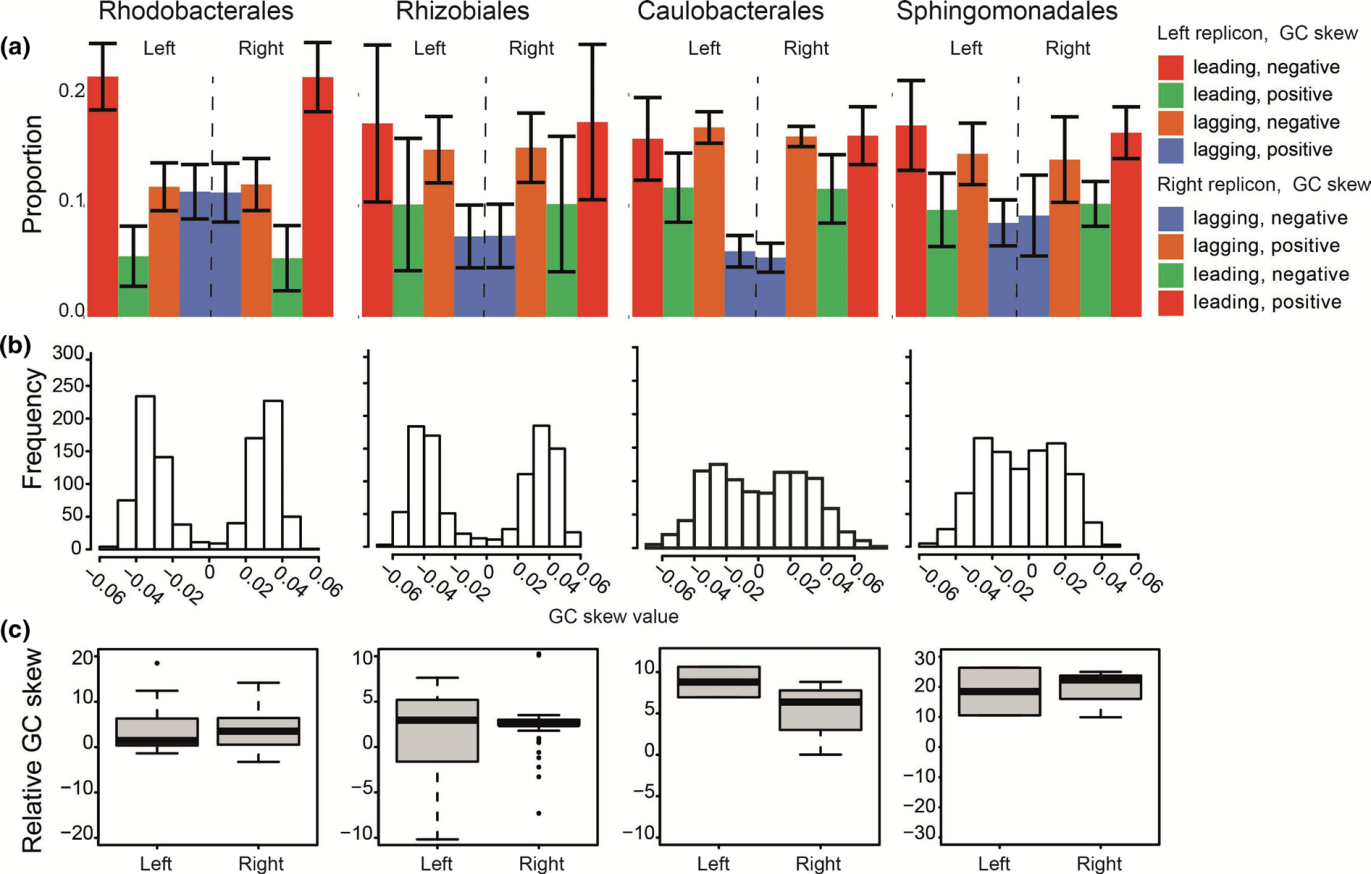
GC skew analyses. (a) Genes located on the leading or lagging strand following the typical GC skew (positive or negative on the right or left replichore, respectively). The data are plotted as the percentage with the standard deviation. (b) Frequency of GC skew mean of all genomes divided into 1000 parts clockwise beginning from *ori*. (c) GC skew of the GTA gene clusters relative to their respective ‘host’ genome [(GC skew_GTA_−GC skew_host_)/GC skew_host_]. The genome was divided into left and right replichores as the values of the GTAs on the right or left replichore would otherwise equalize.

The MCP gene was found on the leading strand of DNA replication in all but five genomes of the Rhodobacterales and Rhizobiales, irrespective of the replichore the cluster was located on (Fig. S3). DNA replication and transcription occur simultaneously in bacteria and head-on collisions between the replisome and RNA polymerase lead to disruptions of both processes that require conflict resolution mechanisms [38]. This has a strong effect on genome organization and evolution because conflicts occur more often on the lagging strand (head-on), and genes oriented this way have higher mutation rates [39]. These disruptions are less common for genes on the leading strand (co-directional) [40–42] and slower evolving core genes tend to have this orientation [41, 43]. Therefore, the GTA genes are like core genes with respect to gene orientation on the chromosome and although recombination events switch GTA gene clusters between replichores, the orientation of the clusters on the leading strand of DNA replication is maintained.

**Fig. 3.**
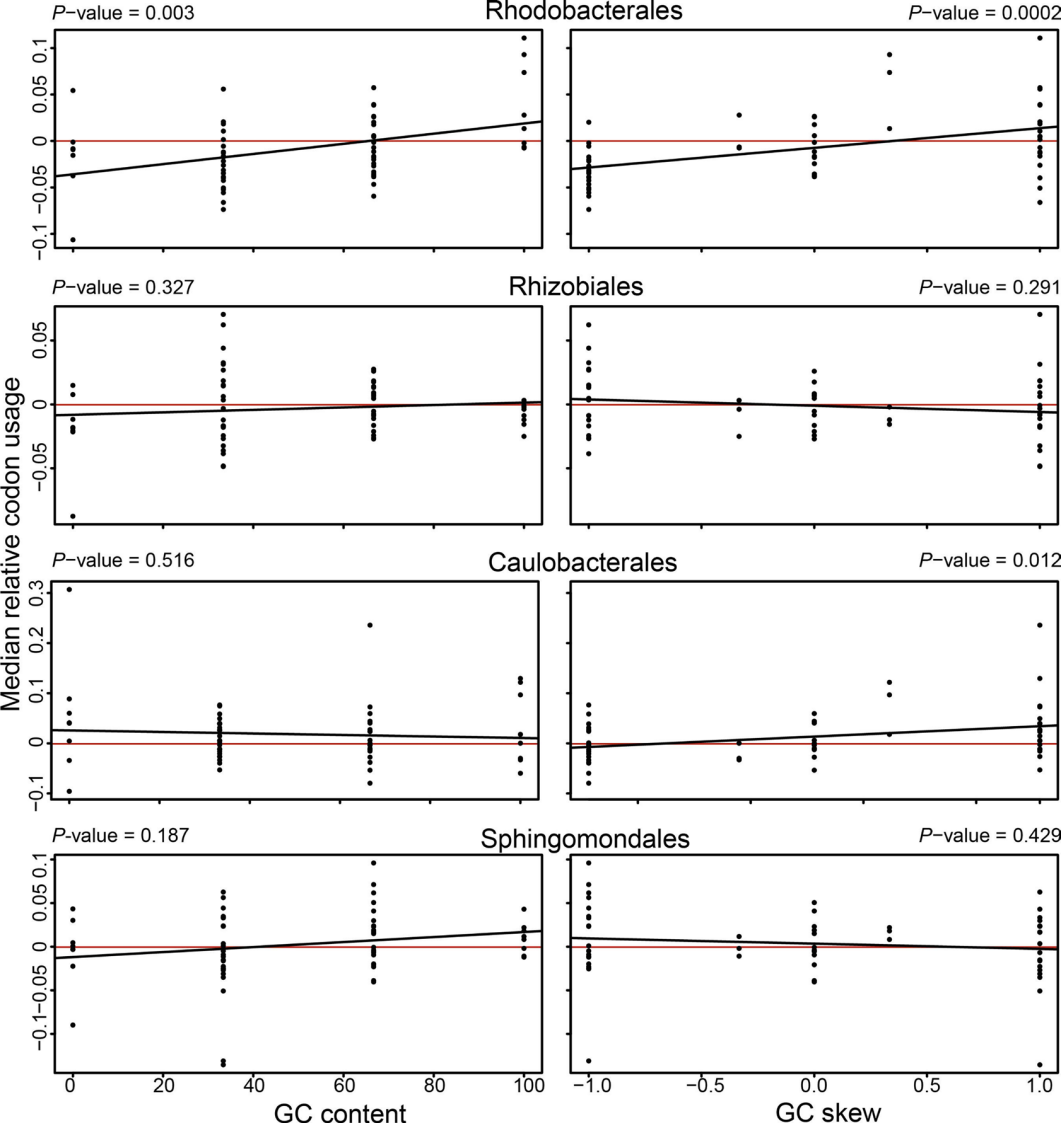
Relationships between codon usage and GC content or GC skew. The median relative codon usages for each order were calculated from the mean codon usage values per genome (peak−non-peak/non-peak) and correlated with the GC content (left) or GC skew (right). The Spearman correlation was used to test significance and *P*-values are given above the plots.

### Cumulative genomic GC skew reveals a unique pattern associated with Rhodobacterales GTA clusters

A typical GC skew pattern for a circular genome is positive on the right replichore and negative on the left replichore. It has been hypothesized that inversions from the co-directional to head-on orientation can be traced by a sign change of the GC skew (e.g. right replichore, leading strand, positive GC skew to right replichore, lagging strand, negative GC skew) (Table 3) [41]. We applied this categorization to all genes whereby not following the typical GC skew indicates an inversion from the other strand. By determining the proportion of genes present in the different genome orientations and with different GC skews we found that co-directional localization was predominant in all four orders, with 8–10 % more genes located on the leading strand than on the lagging strand (Fig. S4a). The overall proportion of genes that follow the typical GC skew was similar in the four orders and ranged from 62.6–66.8 % (Fig. S4b). This trend was most pronounced on the leading strand in the Rhodobacterales (Fig. 2a) and on the lagging strand in the Rhizobiales and Sphingomonadales, while equal numbers of genes followed the typical GC skew on the leading and lagging strands in the Caulobacterales. Approximately equal numbers of genes that follow the typical GC skew or are inverted were found on the lagging strand in the Rhodobacterales (Fig. 2a). Thus, the Rhodobacterales genomes have the strongest conservation of the typical GC skew pattern on the leading strand but the lowest conservation on the lagging strand. The same distribution pattern, although with a slightly stronger preference for the leading strand, was found for core genes (Fig. S4c). The differences between the gene proportions (Fig. S4c) was significant in all orders (Kruskal–Wallis rank sum test *P*-values: Rhizobiales, <2.2×10^−16^; Rhodobacterales, <2.2×10^−16^; Caulobacterales, 3.8×10^−8^; Sphingomondales, 1.3×10^−11^) and also when only core genes were considered (Kruskal–Wallis rank sum test *P*-values: Rhizobiales, <2.2×10^−16^; Rhodobacterales, <2.2×10^−16^; Caulobacterales, 2.2×10^−6^; Sphingomondales, 1.8×10^−8^). Thus, overall, the majority and similar proportions of genes follow the typical GC skew in all four orders. However, these are mainly located on the leading strand in the Rhodobacterales and on the lagging strand or equally distributed in the other three orders, indicating a distinct gene orientation trend among the Rhodobacterales.

**Table 3.** Definitions and characteristics of terms related to GC skew and inversions

Replichore	Strand	Orientations of transcription and translation	GC skew	Typical GC skew^∗^	Potential inversion^†^
Right	Leading +	Co-directional	Positive	Yes	No
Right	Lagging -	Head-on	Negative	No	Yes
Right	Leading -	Co-directional	Negative	No	Yes
Right	Lagging +	Head-on	Positive	Yes	No
Left	Leading +	Co-directional	Positive	No	Yes
Left	Lagging -	Head-on	Negative	Yes	No
Left	Leading -	Co-directional	Negative	Yes	No
Left	Lagging +	Head-on	Positive	No	Yes

*Positive on the right replichore and negative on the left replichore.

†Those genes with GC skews that do not follow the typical GC skew pattern.

Most organisms’ genomes follow the typical GC skew pattern, and it is considered an archetypal genomic property. A high GC skew might reflect the original ancestral genome of the bacteria considered here. Indeed, it was recently proposed that a deviation from this pattern could be used to detect misassembled genomic sequences [42]. In the Rhodobacterales the expected skew is reduced on the lagging strand due to increased inversions, with genes changed from co-directional to head-on orientation. It is unclear why this pattern is found for this group, but it has been suggested that switching genes from co-directional to the head-on orientation might have benefits by increasing evolvability [41], although this is still under debate [44, 45]. There are three hypotheses on the evolutionary history of genome architecture [41] whereby there is either a reduction of head-on genes, a reduction of co-directional genes, or the original ratio is retained. While all four orders studied here have similar proportions of head-on and co-directional genes (Fig. 2a), the inversions on the leading and lagging strands change in different directions in the Rhodobacterales compared to the other three orders, making it difficult to draw a general conclusion from our analysis.

Next, we used cumulative representations of the GC skew (determined over a sliding window) to evaluate the magnitudes of and patterns in the GC skews. The maximal deviations from zero were similar in all four orders, but the patterns varied among them (Fig. 2b). The GC skew values deviated >±0.03 in the Rhodobacterales and Rhizobiales, resulting in clear bimodal distributions (Fig. 2b). In contrast, the distributions were closer to normal in the Sphingomonadales and Caulobacterales, although some bimodality could still be seen. The GTA gene clusters were predominantly located within GC skew peaks in all four orders (Fig. 2c). Indeed, comparing the absolute GC skews of the regions where GTA gene clusters are located to the remainders of the genomes showed that they have greater GC skews than average (Fig. 2c). Interestingly, the strongest deviations could be observed in the Sphingomonadales and Caulobacterales, which have more genes with GC skews around zero (Fig. 2b). This indicates that GTA clusters tend to have high GC skews, irrespective of the rest of the genome’s overall properties.

### Correlation between GC skew and codon usage in the Rhodobacterales

A difference in GC skew value could affect the codon usage, so we examined which codons are enriched in genes with a high GC skew and checked for potential differences in codon usage between genes in GC skew peak and non-peak regions. In the Rhodobacterales we found that codons that were overrepresented in GC skew peaks also had a significantly higher GC content (Fig. 3). This contradicts findings of a negative correlation between GC content and composition bias [25]. Although the Rhizobiales and Rhodobacterales displayed very similar bimodal GC skew distributions, no differences between the codon usage in peak and non-peak regions were observed in the Rhizobiales. Similarly, no significant correlations were found for the other two orders. Next, we compared codon usage to codon GC skew. Significant positive correlations were found for the Rhodobacterales and Caulobacterales (Spearman’s correlation coefficient *P*-values=0.0002 and 0.012, respectively) (Fig. 3). Hence, in the Caulobacterales GC content and in the Rhodobacterales the GC content and GC skew positively correlate with codon usage, which suggests that special codons are used in GC skew peaks and that there is a distinct evolutionary process occurring in the Rhodobacterales.

**Fig. 4.**
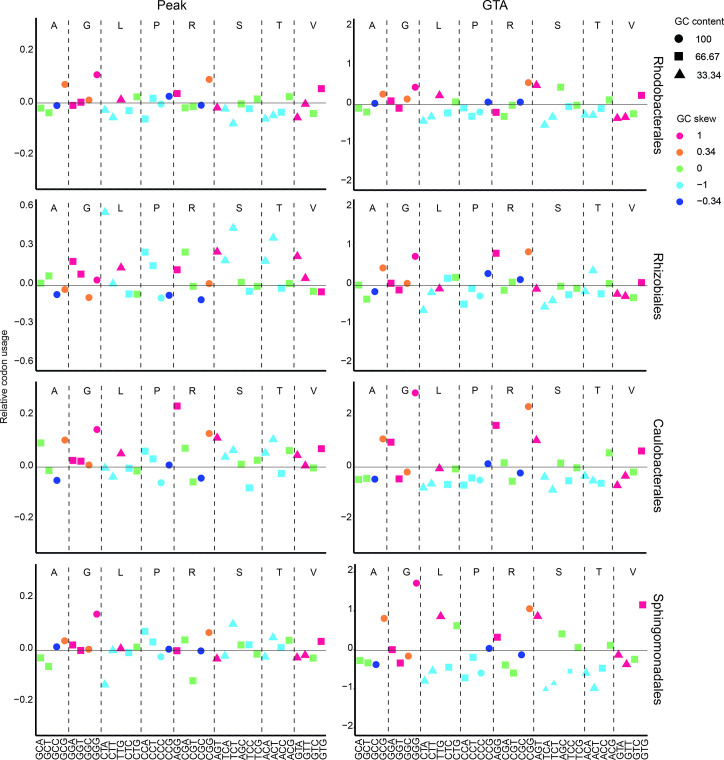
Relative codon usage in GC skew peak genes and GTA genes. Only codons that have a GC content greater than zero and codons specifying a particular amino acid that have the same GC content were considered. The relative codon usage values for peak (or GTA) versus non-peak genes were calculated as [peak (or GTA)]/non-peak. The GC content and skew for each codon in each order are indicated based on their shape and colour, respectively, according to the legend at the top right. The codons are indicated at the bottom of the plots with dashed vertical lines separating the different amino acids, which are indicated above the plots using their one-letter codes.

To determine what influence GC content and GC skew might have on codon usage, we selected codons with identical GC content that encode the same amino acid and compared them based on their GC skew and usage (Fig. 4). We found that codons with higher GC skew were more predominant in GC skew peaks for the Rhodobacterales and Caulobacterales. Thus, in GC skew peaks of those orders, codons with a higher proportion of guanine are preferred instead of cytosine. This was also true for GTA genes compared to non-peak genes in all four orders (Fig. 4). It was previously shown that GTA genes preferentially encode amino acids with a lower carbon content [16], which results in increased GC content [3]. However, our results show that these genes also preferentially use codons with increased GC skew, which is responsible for the location of GTA gene clusters in GC skew peak regions.

Nucleotide strand bias is generally attributed to cytosine deamination events that produce thymine [23, 46]. Therefore, cytosine levels should decrease as they are converted into thymine if deamination is responsible for the GC skew. To see whether there is a lower occurrence of cytosine in the GC skew peaks, we examined codons for their G and C content and their relative usage. We found that an increased codon usage correlated with an increased G content in the Rhodobacterales and Caulobacterales, while the C content tended to decrease (Fig. S5). In the GTA genes, the use of both guanine and cytosine correlated positively with the increase in codon usage (Fig. S6). There was no significant change in guanine or cytosine levels in the peak versus non-peak regions of the Sphingomonadales or Rhizobiales. This could mean that deamination processes are not responsible for the GC skew or at least that they play a smaller role than the preferred selection for guanine-containing codons in the Rhodobacterales and Caulobacterales.

**Fig. 5.**
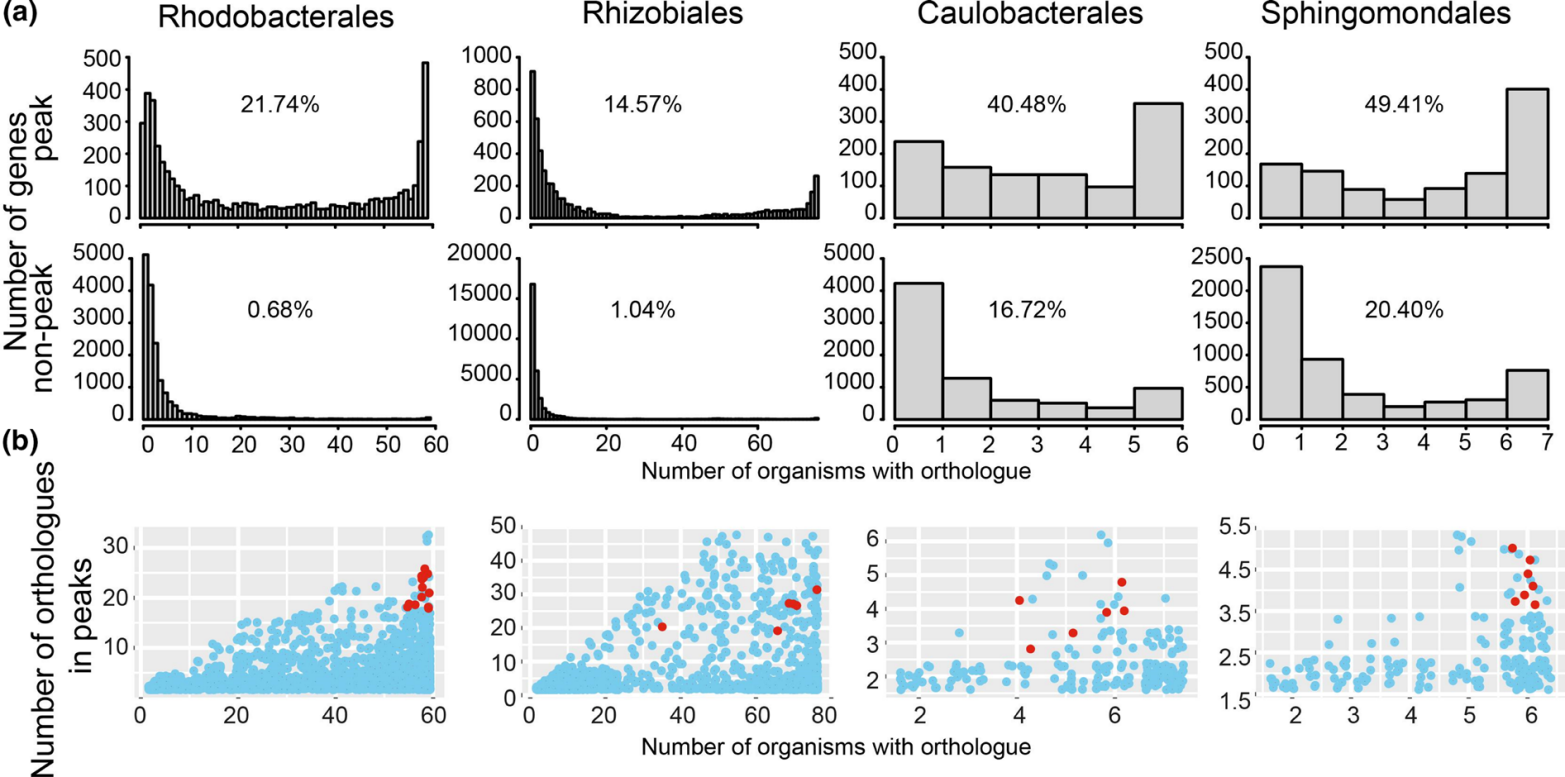
Gene conservation in GC skew peak and non-peak locations. (a) Number of orthologues in and outside of GC skew peaks. The percentage of genes found in ≥90 % of genomes in the orders Rhodobacterales and Rhizobiales or six and five genomes of the Caulobacterales and Sphingomonadales, respectively (representing the numbers of genomes closest to 90%), is shown inside the plot. (b) Number of genomes in which an orthologue was found compared to how often the genes were in GC skew peaks. GTA gene cluster genes are indicated in red.

**Fig. 6.**
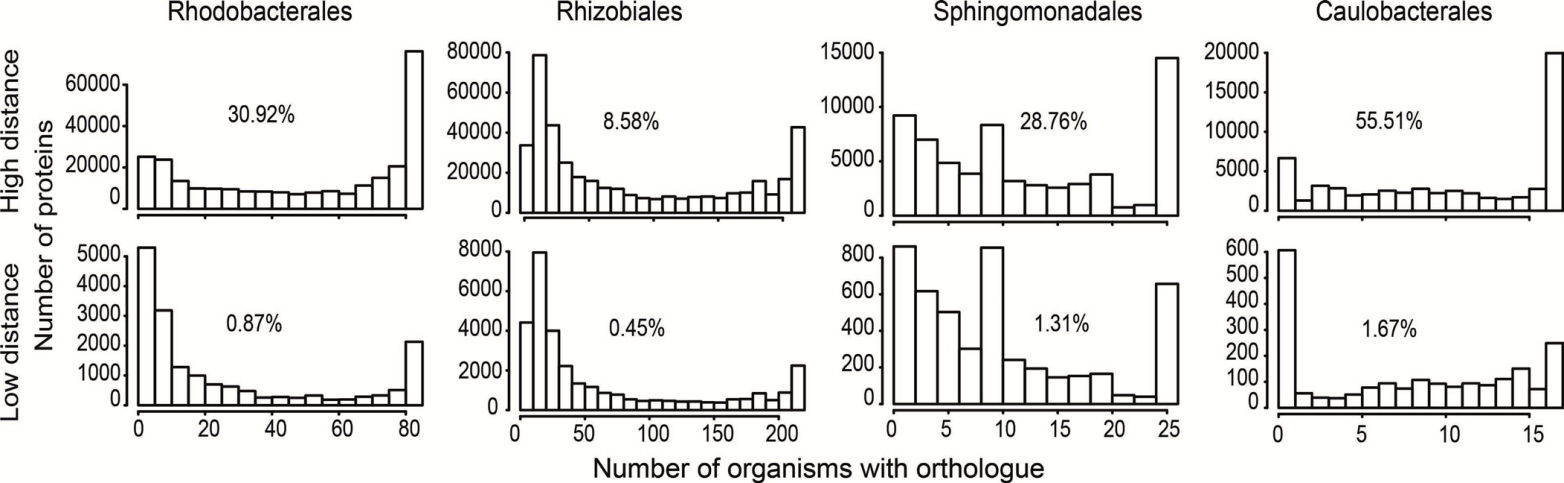
Relationship of gene conservation to distance from repeats. Genes were classified as far from >1/1000 of the genome size (approximately >4 kb for most organisms), or near to <1/1000 of the genome size (approximately <4 kb for most organisms), repeats. The percentages of the number of genes found in ≥90 % of the genomes are shown for the Rhodobacterales and Rhizobiales. The percentages of genes found in more than six or eight genomes (representing the numbers of genomes closest to 90%) are shown for the Caulobacterales and Sphingomonadales, respectively.

### Core and GTA genes are commonly found in GC skew peaks

We found that core genes, defined as those present in ≥90 % of all genomes of an order, were accumulated in regions with GC skew peaks in all four orders (Fig. 5a). The ratios of core genes in peak to non-peak regions (calculated as percentage peak/percentage non-peak) were the most extreme in the Rhodobacterales (32) and Rhizobiales (14), but were also >1 in the Sphingomonadales (2.4) and Caulobacterales (2.4).

To investigate which genes are in GC skew peaks and how conserved their presence in peaks is, we compared the numbers of members of gene families found inside and outside of peaks (without differentiation according to the direction of the peaks) (Fig. 5b). Besides the GTA gene cluster genes that are strongly enriched in GC skew peaks in all four orders, there were multiple examples of genes associated with central physiological processes, such as protein processing (i.e. chaperones; *dnaK* and *clpB*), translation (ribosomes), cell division, nicotinamide adenine dinucleotide metabolism, flagellar motility and recombination, that were frequently found in GC skew peaks (Table S2). Overall, although core genes do not necessarily have to be in GC skew peaks, they are overrepresented in these peaks.

The localization of GTA gene clusters as well as many core genes in genomic areas with GC skew peaks was especially evident for the Rhodobacterales and Rhizobiales, which also have the most pronounced GC skews of the considered orders. This is particularly interesting for two reasons. First, these core genes have been conserved in a wide range of genomes over a long period of time and thus originate from a common ancestor [44, 47]. Second, the GTA gene cluster is believed to have evolved from a prophage that integrated into the genome of a shared ancestor of multiple alphaproteobacterial orders [2], which means that it has been present in these genomes as long as many of the core genes [14] and also shares the property of being preferentially located in GC skew peaks. Thus, it is possible that the original ancestor of these bacteria had a more extreme GC skew, which is supported by the following points. GTAs are evolutionarily related to phages, which have been shown to integrate into the host genome in ways that maximize their success of replication, such as preferential integration on the leading strand and closer to *ter*, and with counter-selection for motifs that could result in disruption of macrodomain structures of the host genome (e.g. motifs associated with the *ter* macrodomain are not found in prophages) [45]. The GTA gene clusters showed similar trends (co-directional orientation and localization closer to *ter*), and possibly these properties have been maintained since evolving from the original prophage. Regarding GC skew, however, prophages and the GTA gene cluster differ. We found no preferential localization of phages in GC skew peaks (Fig. S7), which also fits with their place as accessory mobile genetic elements as opposed to core genes. Moreover, our results have some commonality with findings for eukaryotes, in which highly conserved genes are also contained in genomic regions with strong GC skew [48–50], and it was also shown that those highly conserved genes encode proteins with longer half-lives [50].

**Fig. 7.**
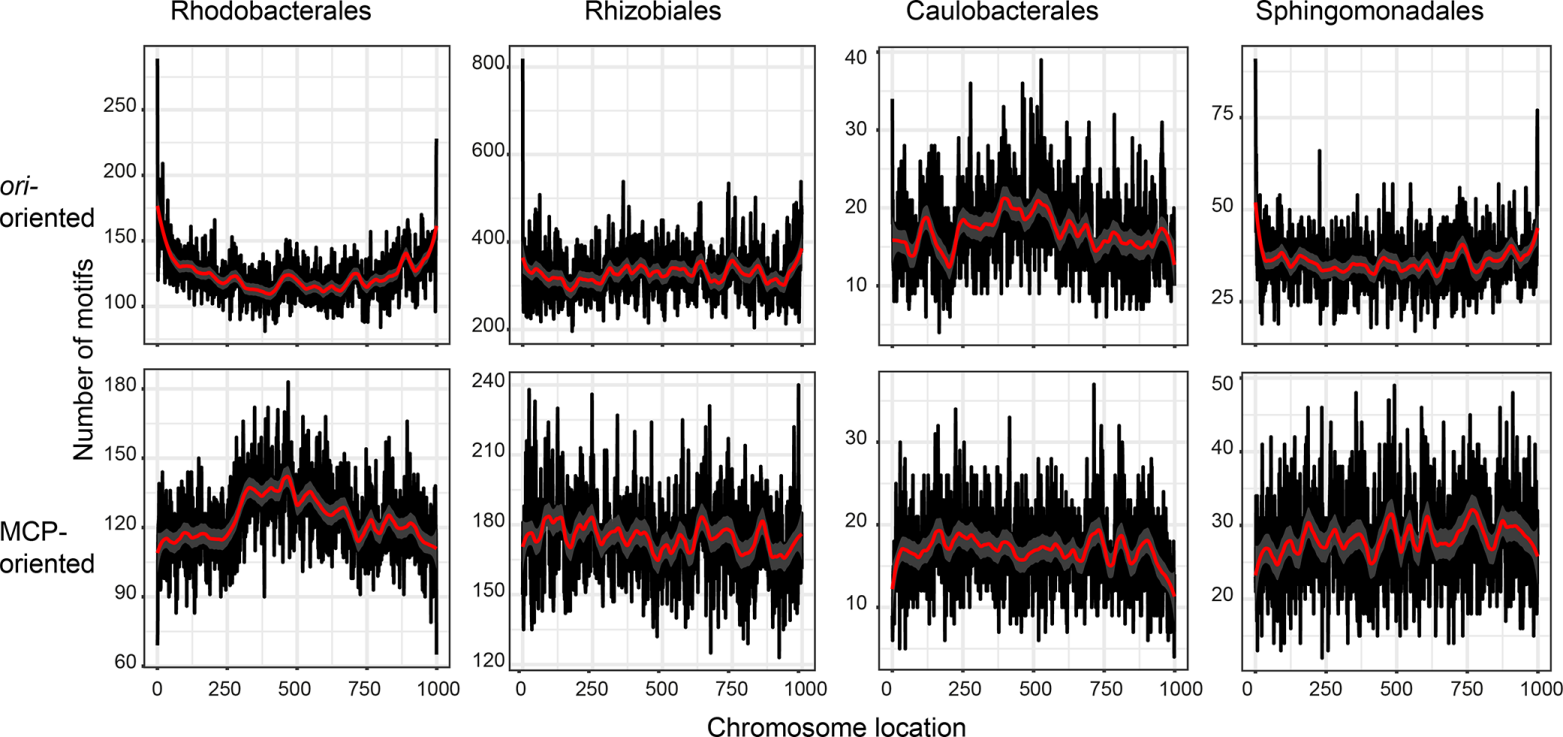
CcrM GANTC methylation motif occurrence across chromosomes. The cumulative occurrence of the methylation motif on chromosomes is plotted with chromosomes normalized to a length of 1000. The genomes were either oriented with position 0 and 1000 representing the origin of replication (ori, top) or the GTA major capsid protein gene (MCP, bottom). A LOESS curve was fitted to visualize local trends (red). The normalized genomes were divided into 100 parts in which the occurrences of the GANTC motif were quantified, with the cumulative number of motifs plotted.

### Core and GTA genes are located far from repetitive elements

Although GTA gene clusters are occasionally translocated between replichores (Figs S2 and 3), the cluster itself and its orientation relative to the direction of DNA replication are well preserved. Therefore, we investigated the stability of these localizations. Regions with repeats, especially long repeats (>800 bp), represent hotspots for chromosome rearrangement and thus increase the local genome plasticity. We found that the distance of GTA gene clusters to long repeats is higher than for most other genes (Table S3). To investigate whether this characteristic is also shared by core genes, we sorted all genes into two groups according to their distances from the nearest repeat, with ‘far’ being considered as >1/1000 of the genome size (approximately >4 kb for most organisms) and ‘near’ being considered as <1/1000 of the genome size (approximately <4 kb for most organisms). Core and GTA genes were predominantly found further away from repeats in all four orders (Fig. 6) and therefore are localized in regions with lower plasticity. Interestingly, a previous analysis of genomes from different phyla showed that genomes with a stronger overall GC skew tend to have fewer repeats and it was concluded that stable chromosomes have higher GC skews and fewer repeats [29]. As discussed above, it was previously documented that GTA genes have a higher GC content [3], and high-GC-content regions have also been shown to have lower rearrangement frequencies [16]. Studies on the gammaproteobacterium *Vibrio parahaemolyticus* and the epsilonproteobacterium *Helicobacter pylori* also showed that GC content and plasticity regions are negatively correlated [51, 52]. This could also contribute to the conserved GTA gene cluster localization.

### Relationship between potential DNA methylation sites and GTA gene cluster localization

One of the key RcGTA regulators is the response regulator protein CtrA [7]. This regulator is almost universally conserved throughout the Alphaproteobacteria [53], with shared and unique roles in different lineages and acting as a key cell cycle regulator in some. In *Caulobacter crescentus *, where CtrA has been best characterized, the *ctrA* promoter and multiple promoters of the CtrA regulon are targeted by the methyltransferase CcrM and the transcriptional regulator GcrA. CcrM methylates the adenine residue of the motif 5′-GANTC-3′, which the protein GcrA then binds to and recruits RNA polymerase to initiate transcription from the associated promoter [54, 55]. While it is well documented that methylation has a strong influence on the CtrA phosphorelay and its regulon, and that CtrA controls the GTA gene cluster in Rhodobacterales members, a connection between all three components (CtrA, methylation by CcrM and the GTA gene cluster) has not yet been investigated to our knowledge. Therefore, we analysed potential CcrM methylation patterns by determining the occurrence of the GANTC recognition motif over the length of the chromosomes (normalized to 1000 bp). A strong increase in methylation motifs in the region around *ori* was observed in all four orders (Fig. 7). Strong and slight additional increases in GANTC motif numbers towards *ter* also exist in the Caulobacterales and Rhodobacterales, respectively.

The reorientation of each chromosome, such that the position of the MCP was the first gene, showed greater overall fluctuations in the GANTC motif pattern occurrences (Fig. 7), probably due to the greater variability in the localization of the GTA genes compared to *ori* (Fig. 1). A slight drop in GANTC numbers at the MCP gene can also be seen in the Caulobacterales and Sphingomonadales genomes. In the Rhodobacterales, this reorientation showed that the GTA regions had the lowest methylation potentials across genomes, even though there is a slight increase in motifs near *ter* in this group. The strongest conservation of GTA gene cluster localization is also found in this order, where it is biased towards *ter*. This is in accordance with our previous study where the Rhodobacterales *ctrA* gene was also found conserved near to *ter* and showed the lowest number of GANTC motifs in *ctrA* promoter regions [56]. Hemi-methylation of this motif during replication has been found to be a signal for transcriptional activation [57]. The presence of less GANTC sequences and its location near *ter* might indicate that *ctrA* transcriptional control is uncoupled from replication in this group. However, future studies are needed to show the potential significance of the low number of GANTC motifs in the GTA gene cluster region.

## Conclusions

In this study, we performed a comprehensive genome structure analysis for four orders of the class Alphaproteobacteria to examine patterns of the RcGTA-type gene cluster localization, genomic GC skew and DNA methylation. We found that the GTA gene cluster shares properties with core genes, such as localization in low-plasticity regions, gene orientation on the leading strand of DNA replication and localization in regions of especially strong GC skew. These high-GC-skew regions at least partly arise due to a selection for codons with higher GC skew and are not necessarily associated with codon GC content enrichment. The GTAs studied here are proposed to have evolved from a phage that integrated into the genome of an ancestral alphaproteobacterial host. Generally, phages try to mimic their host’s genome structure [58], but we did not find any notable elevation of GC skew among phages and prophages. Therefore, it seems that part of the evolutionary process of becoming a GTA included gaining this elevated GC skew, and this might be connected to other properties these genes have in common with core genes, such as their location in regions distant from repetitive elements.

## Supplementary Data

Supplementary material 1

Supplementary material 2

Supplementary material 3

Supplementary material 4

## References

[1] Lang AS, Westbye AB, Beatty JT (2017). The distribution, evolution, and roles of gene transfer agents in prokaryotic genetic exchange. Annu Rev Virol.

[2] Lang AS, Beatty JT (2007). Importance of widespread gene transfer agent genes in α-proteobacteria. Trends Microbiol.

[3] Shakya M, Soucy SM, Zhaxybayeva O (2017). Insights into origin and evolution of α-proteobacterial gene transfer agents. Virus Evol.

[4] Tomasch J, Wang H, Hall ATK, Patzelt D, Preusse M (2018). Packaging of *Dinoroseobacter shibae* DNA into gene transfer agent particles is not random. Genome Biol Evol.

[5] Biers EJ, Wang K, Pennington C, Belas R, Chen F (2008). Occurrence and expression of gene transfer agent genes in marine bacterioplankton. Appl Environ Microbiol.

[6] Nagao N, Yamamoto J, Komatsu H, Suzuki H, Hirose Y (2015). The gene transfer agent-like particle of the marine phototrophic bacterium *Rhodovulum sulfidophilum*. Biochem Biophys Rep.

[7] Lang AS, Beatty JT (2000). Genetic analysis of a bacterial genetic exchange element: the gene transfer agent of *Rhodobacter capsulatus*. Proc Natl Acad Sci.

[8] Redfield RJ (2014). Do bacteria have sex?. Microbes Evol.

[9] Marrs B, Wall JD, Gest H (1977). Emergence of the biochemical genetics and molecular biology of photosynthetic bacteria. Trends in Biochemical Sciences.

[10] Québatte M, Christen M, Harms A, Körner J, Christen B (2017). Gene transfer agent promotes evolvability within the fittest subpopulation of a bacterial pathogen. Cell Syst.

[11] Redfield RJ, Soucy SM (2018). Evolution of bacterial gene transfer agents. Front Microbiol.

[12] Hynes AP, Mercer RG, Watton DE, Buckley CB, Lang AS (2012). DNA packaging bias and differential expression of gene transfer agent genes within a population during production and release of the *Rhodobacter capsulatus* gene transfer agent, RcGTA. Mol Microbiol.

[13] Fogg PCM, Westbye AB, Beatty JT (2012). One for all or all for one: heterogeneous expression and host cell lysis are key to gene transfer agent activity in *Rhodobacter capsulatus*. PLoS One.

[14] Lang AS, Zhaxybayeva O, Beatty JT (2012). Gene transfer agents: phage-like elements of genetic exchange. Nat Rev Microbiol.

[15] Westbye AB, Beatty JT, Lang AS (2017). Guaranteeing a captive audience: coordinated regulation of gene transfer agent (GTA) production and recipient capability by cellular regulators. Curr Opin Microbiol.

[16] Kogay R, Wolf YI, Koonin EV, Zhaxybayeva O (2020). Selection for reducing energy cost of protein production drives the GC content and amino acid composition bias in gene transfer agents. mBio.

[17] Westbye AB, O’Neill Z, Schellenberg-Beaver T, Beatty JT (2017). The *Rhodobacter capsulatus* gene transfer agent is induced by nutrient depletion and the RNAP omega subunit. Microbiology.

[18] Koppenhöfer S, Wang H, Scharfe M, Kaever V, Wagner-Döbler I (2019). Integrated transcriptional regulatory network of quorum sensing, replication control, and SOS response in *Dinoroseobacter shibae*. Front Microbiol.

[19] Lobry JR (1996). Asymmetric substitution patterns in the two DNA strands of bacteria. Mol Biol Evol.

[20] Freeman JM, Plasterer TN, Smith TF, Mohr SC (1998). Patterns of genome organization in bacteria. Science.

[21] Rocha EPC (2004). Order and disorder in bacterial genomes. Curr Opin Microbiol.

[22] Rocha EPC (2004). The replication-related organization of bacterial genomes. Microbiology.

[23] Bhagwat AS, Hao W, Townes JP, Lee H, Tang H (2016). Strand-biased cytosine deamination at the replication fork causes cytosine to thymine mutations in *Escherichia coli*. Proc Natl Acad Sci.

[24] Kono N, Tomita M, Arakawa K (2018). Accelerated laboratory evolution reveals the influence of replication on the GC skew in *Escherichia coli*. Genome Biol Evol.

[25] Zhao HL, Xia ZK, Zhang FZ, Ye YN, Guo FB (2015). Multiple factors drive replicating strand composition bias in bacterial genomes. Int J Mol Sci.

[26] Achaz G, Coissac E, Netter P, Rocha EPC (2003). Associations between inverted repeats and the structural evolution of bacterial genomes. Genetics.

[27] Vandecraen J, Chandler M, Aertsen A, Van Houdt R (2017). The impact of insertion sequences on bacterial genome plasticity and adaptability. Crit Rev Microbiol.

[28] Sela I, Wolf YI, Koonin EV (2019). Selection and genome plasticity as the key factors in the evolution of bacteria. Phys Rev X.

[29] Rocha EPC, Blanchard A (2002). Genomic repeats, genome plasticity and the dynamics of *Mycoplasma* evolution. Nucleic Acids Res.

[30] Gao F, Zhang C-T (2008). Ori-Finder: a web-based system for finding *oriC*s in unannotated bacterial genomes. BMC Bioinformatics.

[31] Lechner M, Findeiss S, Steiner L, Marz M, Stadler PF (2011). Proteinortho: detection of (co-)orthologs in large-scale analysis. BMC Bioinformatics.

[32] Arndt D, Grant JR, Marcu A, Sajed T, Pon A (2016). PHASTER: a better, faster version of the PHAST phage search tool. Nucleic Acids Res.

[33] Achaz G, Boyer F, Rocha EPC, Viari A, Coissac E (2007). Repseek, a tool to retrieve approximate repeats from large DNA sequences. Bioinformatics.

[34] Kung SH, Retchless AC, Kwan JY, Almeida RPP (2013). Effects of DNA size on transformation and recombination efficiencies in *Xylella fastidiosa*. Appl Environ Microbiol.

[35] Darling ACE, Mau B, Blattner FR, Perna NT (2004). Mauve: multiple alignment of conserved genomic sequence with rearrangements. Genome Res.

[36] Kumar S, Stecher G, Li M, Knyaz C, Tamura K (2018). MEGA X: Molecular Evolutionary Genetics Analysis across computing platforms. Mol Biol Evol.

[37] Hawkey J, Hamidian M, Wick RR, Edwards DJ, Billman-Jacobe H (2015). ISMapper: identifying transposase insertion sites in bacterial genomes from short read sequence data. BMC Genomics.

[38] Mangiameli SM, Merrikh CN, Wiggins PA, Merrikh H (2017). Transcription leads to pervasive replisome instability in bacteria. Elife.

[39] Lin Y-L, Pasero P (2012). Interference between DNA replication and transcription as a cause of genomic instability. Curr Genomics.

[40] Rocha EPC, Danchin A (2003). Gene essentiality determines chromosome organisation in bacteria. Nucleic Acids Res.

[41] Merrikh CN, Merrikh H (2018). Gene inversion potentiates bacterial evolvability and virulence. Nat Commun.

[42] Lu J, Salzberg SL (2020). SkewIT: The Skew Index Test for large-scale GC Skew analysis of bacterial genomes. PLoS Comput Biol.

[43] Liu H, Zhang J (2022). Testing the adaptive hypothesis of lagging-strand encoding in bacterial genomes. Nat Commun.

[44] Dewey CN, Pachter L (2006). Evolution at the nucleotide level: the problem of multiple whole-genome alignment. Hum Mol Genet.

[45] Bobay LM, Rocha EPC, Touchon M (2013). The adaptation of temperate bacteriophages to their host genomes. Mol Biol Evol.

[46] Duncan BK, Miller JH (1980). Mutagenic deamination of cytosine residues in DNA. Nature.

[47] Daubin V, Gouy M, Perrière G (2002). A phylogenomic approach to bacterial phylogeny: evidence of a core of genes sharing a common history. Genome Res.

[48] Hartono SR, Korf IF, Chédin F (2015). GC skew is a conserved property of unmethylated CpG island promoters across vertebrates. Nucleic Acids Res.

[49] Ginno PA, Lim YW, Lott PL, Korf I, Chédin F (2013). GC skew at the 5’ and 3’ ends of human genes links R-loop formation to epigenetic regulation and transcription termination. Genome Res.

[50] Dai Y, Holland PWH (2019). The interaction of natural selection and GC skew may drive the fast evolution of a sand rat homeobox gene. Mol Biol Evol.

[51] Fischer W, Windhager L, Rohrer S, Zeiller M, Karnholz A (2010). Strain-specific genes of *Helicobacter pylori*: genome evolution driven by a novel type IV secretion system and genomic island transfer. Nucleic Acids Res.

[52] Han H, Wong H-C, Kan B, Guo Z, Zeng X (2008). Genome plasticity of *Vibrio parahaemolyticus*: microevolution of the “pandemic group.”. BMC Genomics.

[53] Brilli M, Fondi M, Fani R, Mengoni A, Ferri L (2010). The diversity and evolution of cell cycle regulation in alpha-proteobacteria: a comparative genomic analysis. BMC Syst Biol.

[54] Fioravanti A, Fumeaux C, Mohapatra SS, Bompard C, Brilli M (2013). DNA binding of the cell cycle transcriptional regulator GcrA depends on N6-adenosine methylation in *Caulobacter crescentus* and other Alphaproteobacteria. PLoS Genet.

[55] Haakonsen DL, Yuan AH, Laub MT (2015). The bacterial cell cycle regulator GcrA is a σ^70^ cofactor that drives gene expression from a subset of methylated promoters. Genes Dev.

[56] Tomasch J, Koppenhöfer S, Lang AS (2021). Connection between chromosomal location and function of CtrA phosphorelay genes in Alphaproteobacteria. Front Microbiol.

[57] Mohapatra SS, Fioravanti A, Vandame P, Spriet C, Pini F (2020). Methylation-dependent transcriptional regulation of crescentin gene (*creS*) by GcrA in *Caulobacter crescentus*. Mol Microbiol.

[58] Samson JE, Magadán AH, Sabri M, Moineau S (2013). Revenge of the phages: defeating bacterial defences. Nat Rev Microbiol.

[59] Yin T, Cook D, Lawrence M (2012). ggbio: an R package for extending the grammar of graphics for genomic data. Genome Biol.

[60] Toedling J, Skylar O, Sklyar O, Krueger T, Fischer JJ (2007). Ringo--an R/Bioconductor package for analyzing ChIP-chip readouts. BMC Bioinformatics.

[61] Elek A, Kuzman M, Vlahovicek K (2019). CoRdon: codon usage analysis and prediction of gene expressivity. R package version 140.

