# Supplementary material for: Shared properties of gene transfer agent and core genes revealed by comparative genomics of Alphaproteobacteria
Source: Microb Genom. 2022 Nov 9;8(11):mgen000890. doi: 10.1099/mgen.0.000890 (PMC9836097; doi:10.1099/mgen.0.000890)
Supplement: Supplementary material 1 [file mgen-8-890-s001.pdf]

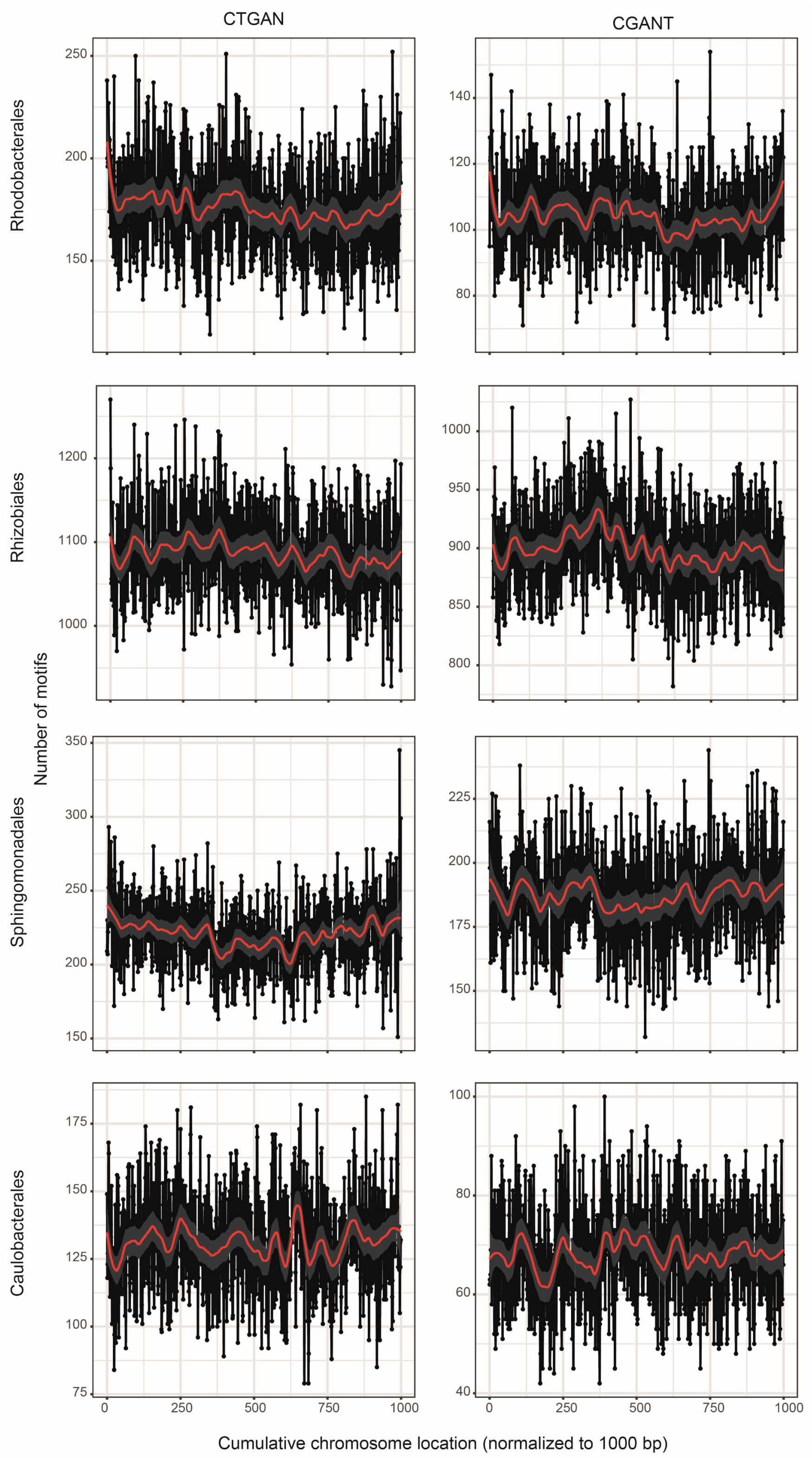

Figure S1. Cumulative display of CTGAN and CGANT motifs. The genome location is normalized to 1000 bp, with positions 0 and 1000 corresponding to *ori*. Smoothed means are displayed with red lines.

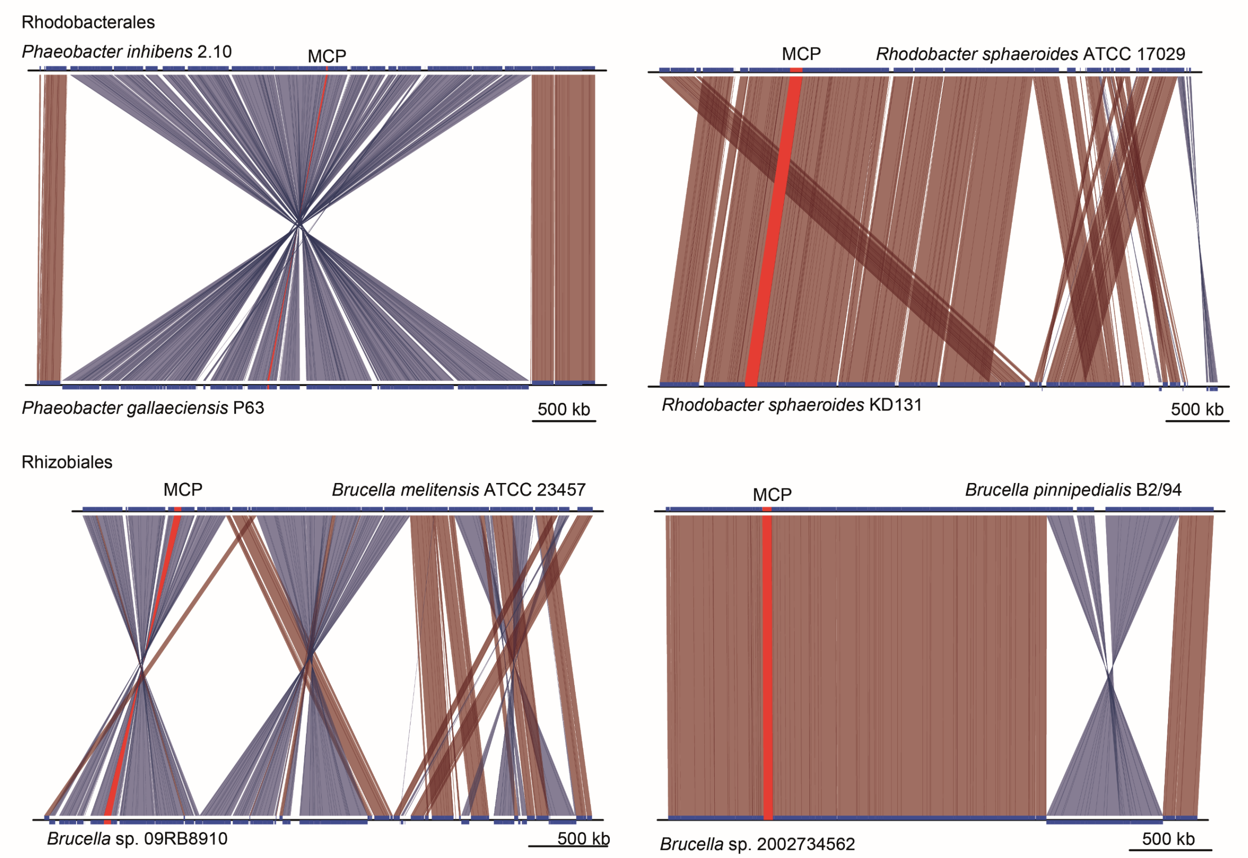

Figure S2. Location of the major capsid protein (MCP) gene of the GTA gene cluster in pairs of closely related organisms in the Rhodobacterales (top) and Rhizobiales (bottom). Inversions of regions of the genomes between the pairs of organisms (left) are visualized in blue and the MCP genes are in red. The blue bars on the top and bottom of each comparison indicate the locations of genes on the genomes of each organism. The left pairs show cases where inversions have transferred the GTA gene clusters to the opposite strand while the right pairs show cases where no inversion of the GTA gene cluster occurred.

A

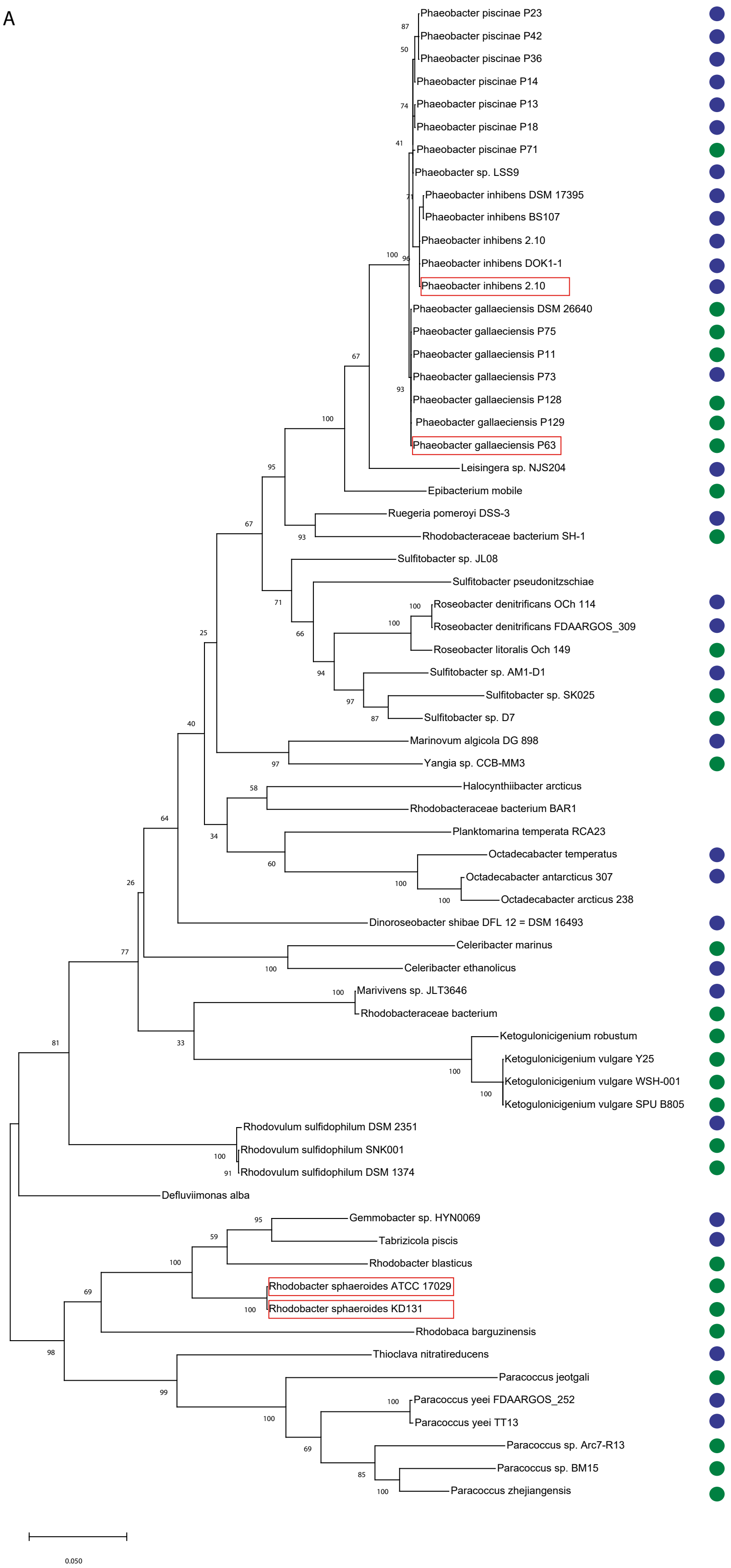
Phaeobacter piscinae P23
Phaeobacter piscinae P42
Phaeobacter piscinae P36
Phaeobacter piscinae P14
Phaeobacter piscinae P13
Phaeobacter piscinae P18
Phaeobacter piscinae P71
Phaeobacter sp. LSS9
Phaeobacter inhibens DSM 17395
Phaeobacter inhibens BS107
Phaeobacter inhibens 2.10
Phaeobacter inhibens DOK1-1
Phaeobacter inhibens 2.10
Phaeobacter gallaeciensis DSM 26640
Phaeobacter gallaeciensis P75
Phaeobacter gallaeciensis P11
Phaeobacter gallaeciensis P73
Phaeobacter gallaeciensis P128
Phaeobacter gallaeciensis P129
Phaeobacter gallaeciensis P63
Leisingera sp. NJS204
Epibacterium mobile
Ruegeria pomeroyi DSS-3
Rhodobacteraceae bacterium SH-1
Sulfitobacter sp. JL08
Sulfitobacter pseudonitzschiae
Roseobacter denitrificans OCh 114
Roseobacter denitrificans FDAARGOS_309
Roseobacter litoralis Och 149
Sulfitobacter sp. AM1-D1
Sulfitobacter sp. SK025
Sulfitobacter sp. D7
Marinovum algicola DG 898
Yangia sp. CCB-MM3
Halocynthiibacter arcticus
Rhodobacteraceae bacterium BAR1
Planktomarina temperata RCA23
Octadecabacter temperatus
Octadecabacter antarcticus 307
Octadecabacter arcticus 238
Dinoroseobacter shibae DFL 12 = DSM 16493
Celeribacter marinus
Celeribacter ethanolicus
Marivivens sp. JLT3646
Rhodobacteraceae bacterium
Ketogulonicigenium robustum
Ketogulonicigenium vulgare Y25
Ketogulonicigenium vulgare WSH-001
Ketogulonicigenium vulgare SPU B805
Rhodovulum sulfidophilum DSM 2351
Rhodovulum sulfidophilum SNK001
Rhodovulum sulfidophilum DSM 1374
Defluviimonas alba
Gemmobacter sp. HYN0069
Tabrizicola piscis
Rhodobacter blasticus
Rhodobacter sphaeroides ATCC 17029
Rhodobacter sphaeroides KD131
Rhodobaca barguzinensis
Thioclava nitratireducens
Paracoccus jeotgali
Paracoccus yeei FDAARGOS_252
Paracoccus yeei TT13
Paracoccus sp. Arc7-R13
Paracoccus sp. BM15
Paracoccus zhejiangensis
0.050

B

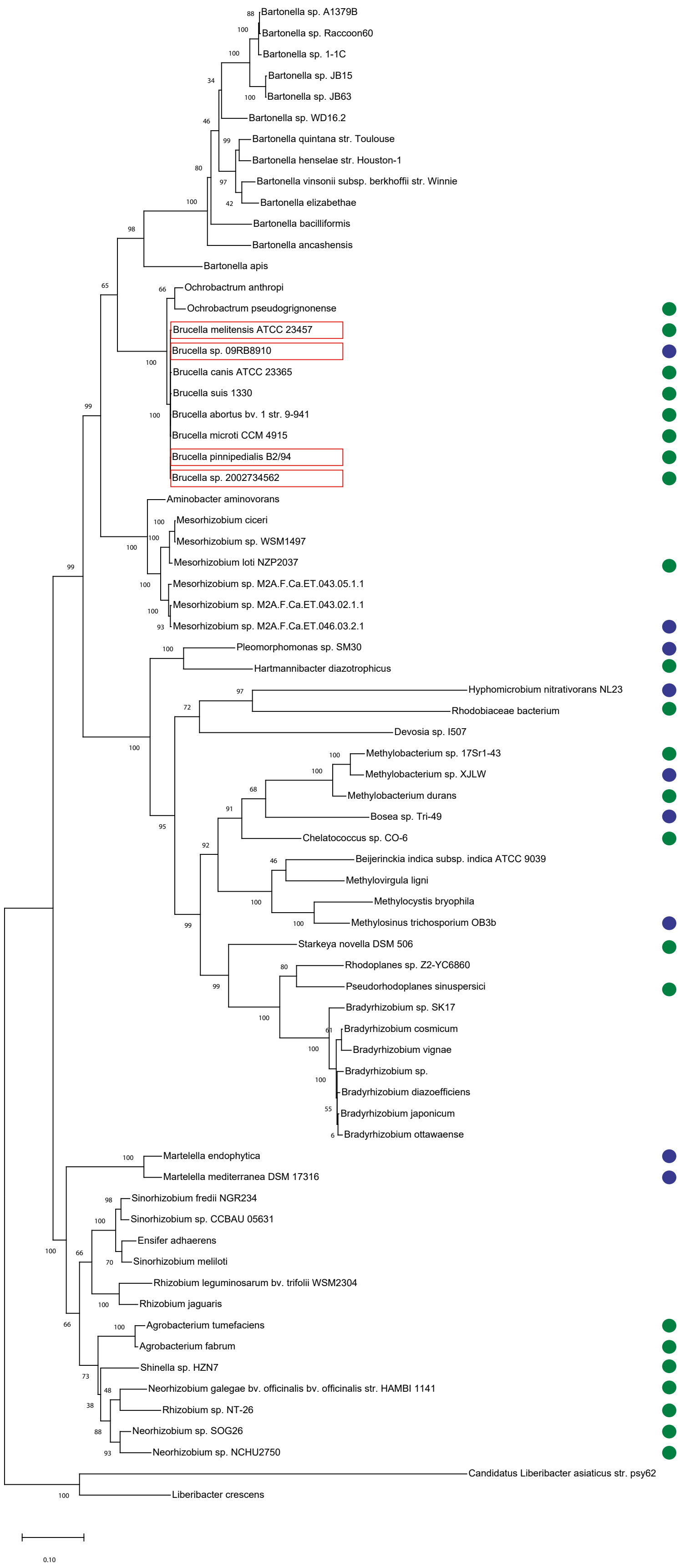
Bartonella sp. A1379B
Bartonella sp. Raccoon60
Bartonella sp. 1-1C
Bartonella sp. JB15
Bartonella sp. JB63
Bartonella sp. WD16.2
Bartonella quintana str. Toulouse
Bartonella henselae str. Houston-1
Bartonella vinsonii subsp. berkhoffii str. Winnie
Bartonella elizabethae
Bartonella bacilliformis
Bartonella ancashensis
Bartonella apis
Ochrobactrum anthropi
Ochrobactrum pseudogrignonense
Brucella melitensis ATCC 23457
Brucella sp. 09RB8910
Brucella canis ATCC 23365
Brucella suis 1330
Brucella abortus bv. 1 str. 9-941
Brucella microti CCM 4915
Brucella pinnipedialis B2/94
Brucella sp. 2002734562
Aminobacter aminovorans
Mesorhizobium ciceri
Mesorhizobium sp. WSM1497
Mesorhizobium loti NZP2037
Mesorhizobium sp. M2A.F.Ca.ET.043.05.1.1
Mesorhizobium sp. M2A.F.Ca.ET.043.02.1.1
Mesorhizobium sp. M2A.F.Ca.ET.046.03.2.1
Pleomorphomonas sp. SM30
Hartmannibacter diazotrophicus
Hyphomicrobium nitrativorans NL23
Rhodobiaceae bacterium
Devosia sp. I507
Methylobacterium sp. 17Sr1-43
Methylobacterium sp. XJLW
Methylobacterium durans
Bosea sp. Tri-49
Chelatococcus sp. CO-6
Beijerinckia indica subsp. indica ATCC 9039
Methylovirgula ligni
Methylocystis bryophila
Methylosinus trichosporium OB3b
Starkeya novella DSM 506
Rhodoplanes sp. Z2-YC6860
Pseudorhodoplanes sinuspersici
Bradyrhizobium sp. SK17
Bradyrhizobium cosmicum
Bradyrhizobium vignae
Bradyrhizobium sp.
Bradyrhizobium diazoefficiens
Bradyrhizobium japonicum
Bradyrhizobium ottawaense
Martelella endophytica
Martelella mediterranea DSM 17316
Sinorhizobium fredii NGR234
Sinorhizobium sp. CCBAU 05631
Ensifer adhaerens
Sinorhizobium meliloti
Rhizobium leguminosarum bv. trifolii WSM2304
Rhizobium jaguaris
Agrobacterium tumefaciens
Agrobacterium fabrum
Shinella sp. HZN7
Neorhizobium galegae bv. officinalis bv. officinalis str. HAMBI 1141
Rhizobium sp. NT-26
Neorhizobium sp. SOG26
Neorhizobium sp. NCHU2750
Candidatus Liberibacter asiaticus str. psy62
Liberibacter crescens
0.10

C

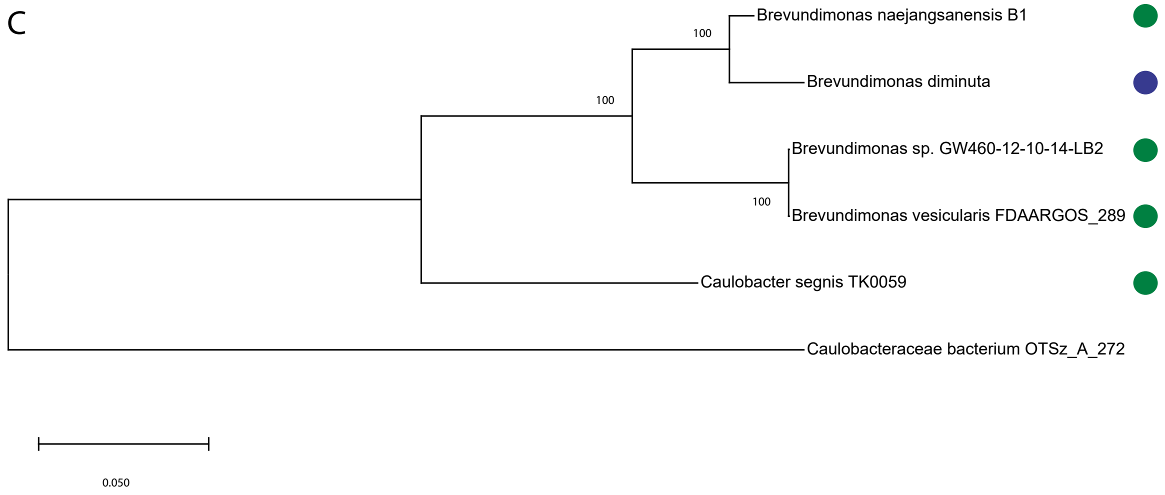
Brevundimonas naejangsanensis B1
Brevundimonas diminuta
Brevundimonas sp. GW460-12-10-14-LB2
Brevundimonas vesicularis FDAARGOS_289
Caulobacter segnis TK0059
Caulobacteraceae bacterium OTSz_A_272
100
100
100
0.050

D

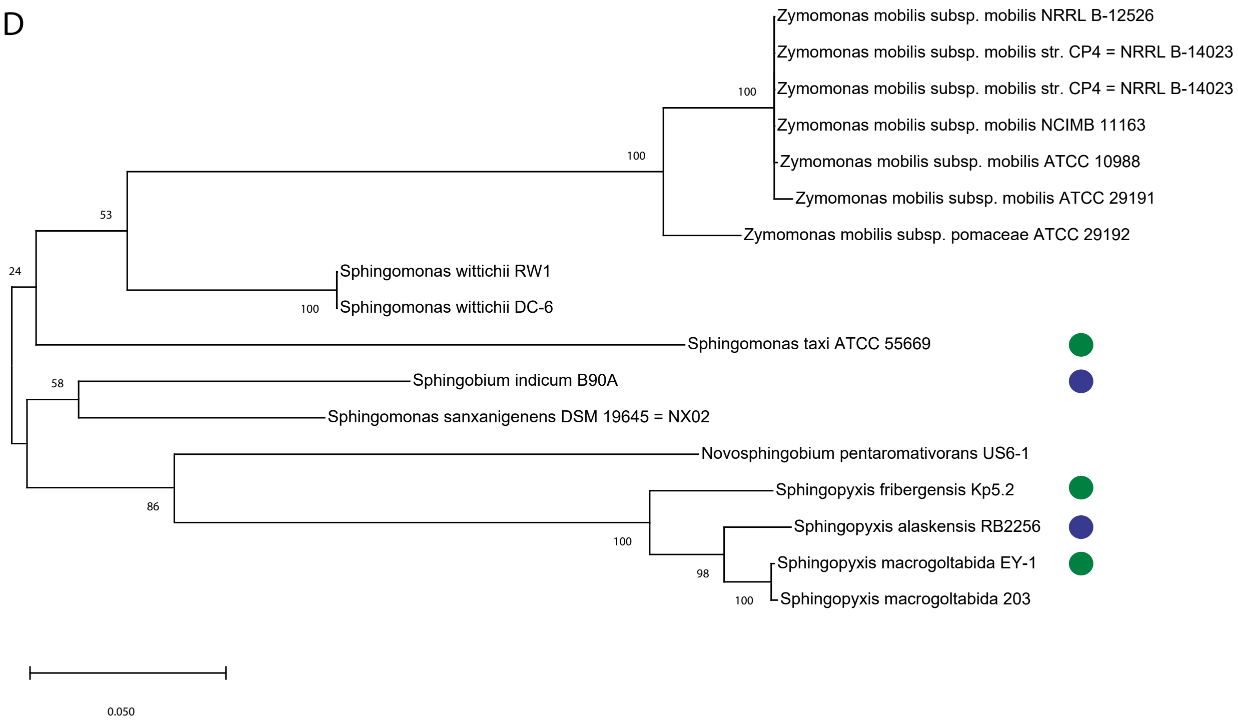
Zymomonas mobilis subsp. mobilis NRRL B-12526
Zymomonas mobilis subsp. mobilis str. CP4 = NRRL B-14023
Zymomonas mobilis subsp. mobilis str. CP4 = NRRL B-14023
Zymomonas mobilis subsp. mobilis NCIMB 11163
Zymomonas mobilis subsp. mobilis ATCC 10988
Zymomonas mobilis subsp. mobilis ATCC 29191
Zymomonas mobilis subsp. pomaceae ATCC 29192
Sphingomonas wittichii RW1
Sphingomonas wittichii DC-6
Sphingomonas taxi ATCC 55669
Sphingobium indicum B90A
Sphingomonas sanxanigenens DSM 19645 = NX02
Novosphingobium pentaromativorans US6-1
Sphingopyxis fribergensis Kp5.2
Sphingopyxis alaskensis RB2256
Sphingopyxis macrogoltabida EY-1
Sphingopyxis macrogoltabida 203
100
100
53
24
100
58
86
100
98
100
0.050

Figure S3. Phylogenetic trees based on RpoB amino acid sequences for the four investigated orders of Alphaproteobacteria. A. The Rhodobacterales. B. The Rhizobiales. C. The Caulobacterales. D. The Sphingomondales. Blue circles indicate GTA gene cluster location on the left replichore and green circles indicate its location on the right replichore. The Rhodobacterales and Rhizobiales organisms that were used for the Figure S2 alignments are outlined in red. RpoB sequences were identified with ProteinOrtho using sequences AAV96733.1, AAN30162.1, ANF54622.1 and ABF53199 as references for the Rhodobacterales, Rhizobiales, Caulobacterales, and Sphingomonadales, respectively. The alignments and trees were generated with MEGAX. The default settings were used for pairwise and multiple alignments. Partial deletion with a delay divergent cutoff of 30% was used for gaps and missing data. The trees were constructed with the maximum-likelihood method. The branching patterns were evaluated using 100 bootstrap replicates, and the LG model was applied with gamma distribution at invariant sites. The site coverage cutoff was 95%.

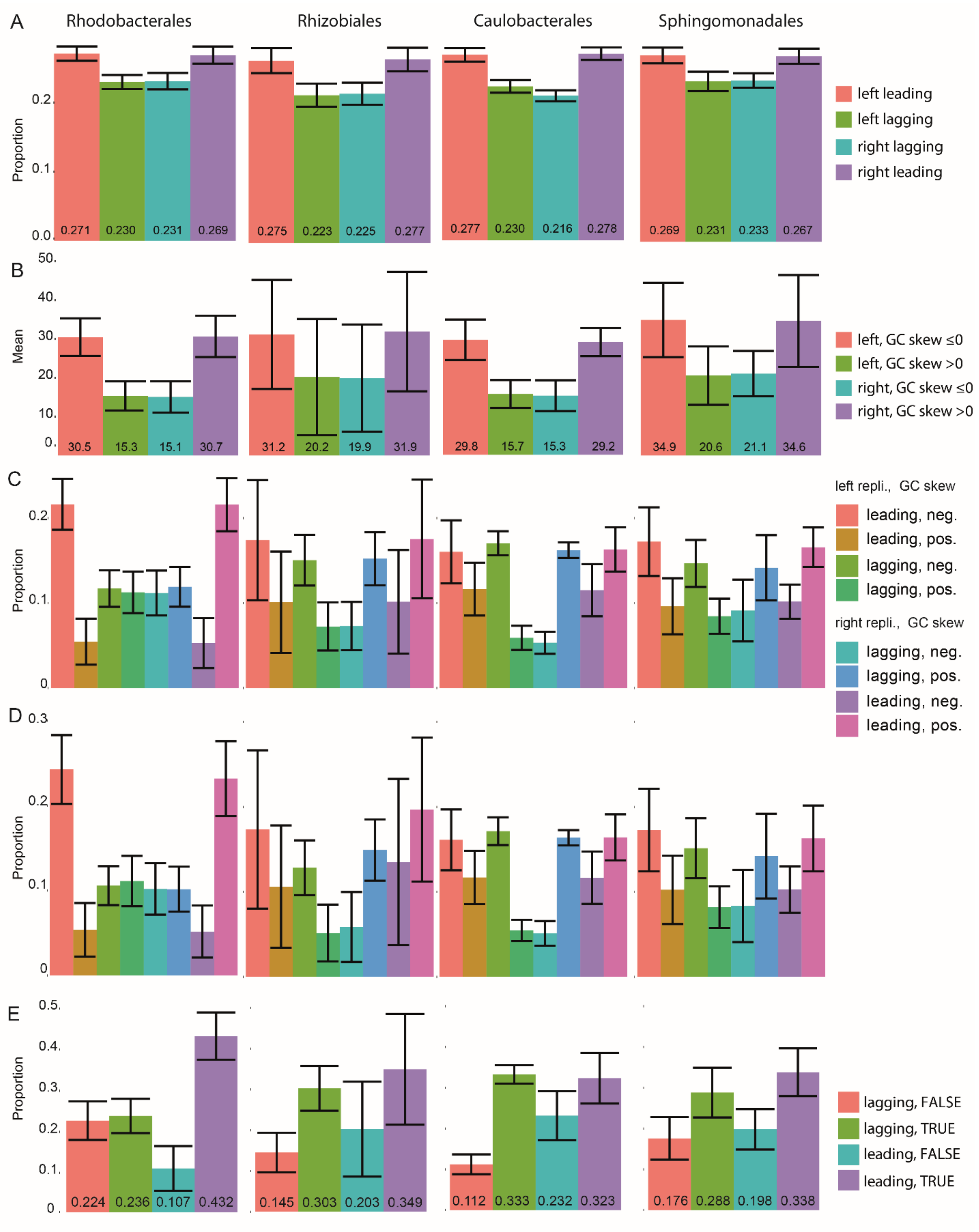
A
Rhodobacterales
Rhizobiales
Caulobacterales
Sphingomonadales
Proportion
0.0
0.1
0.2
0.271 0.230 0.231 0.269
0.275 0.223 0.225 0.277
0.277 0.230 0.216 0.278
0.269 0.231 0.233 0.267
left leading
left lagging
right lagging
right leading
B
Mean
0
10
20
30
40
50
30.5 15.3 15.1 30.7
31.2 20.2 19.9 31.9
29.8 15.7 15.3 29.2
34.9 20.6 21.1 34.6
left, GC skew ≤0
left, GC skew >0
right, GC skew ≤0
right, GC skew >0
C
Proportion
0
0.1
0.2
left repli., GC skew
leading, neg.
leading, pos.
lagging, neg.
lagging, pos.
right repli., GC skew
lagging, neg.
lagging, pos.
leading, neg.
leading, pos.
D
Proportion
0
0.1
0.2
0.3
E
Proportion
0
0.1
0.2
0.3
0.4
0.5
0.224 0.236 0.107 0.432
0.145 0.303 0.203 0.349
0.112 0.333 0.232 0.323
0.176 0.288 0.198 0.338
lagging, FALSE
lagging, TRUE
leading, FALSE
leading, TRUE

Figure S4. Gene localizations and properties in relation to replichores and direction of DNA replication. Standard deviations are represented by the error bars. A. Proportion of genes that are localized on the two replichores on the positive or negative strands with respect to the direction of DNA replication. The leading strand corresponds to the positive strand on the right replichore and the negative strand on the left replichore. The different groups are colored as indicated in the legend. B. Percentages of genes with positive or negative GC skew on the right or left replichores. The different affiliations are colored as indicated in the legend. The exact values are given inside the respective bars. C. Proportions of genes located on the leading and lagging strands with positive or negative GC skews. The different affiliations are colored as indicated in the legend. D. Proportions of core genes located on the leading and lagging strands with positive or negative GC skews. The color legend is the same as for panel C. The exact values are given within the respective bars. E. Genes located on the leading or lagging strand following the classical GC skew (positive or negative on the right or left replichore, respectively) are indicated with TRUE, while those not (negative or positive on the right or left replichore, respectively) are indicated with FALSE. The different affiliations are colored as indicated in the legend.

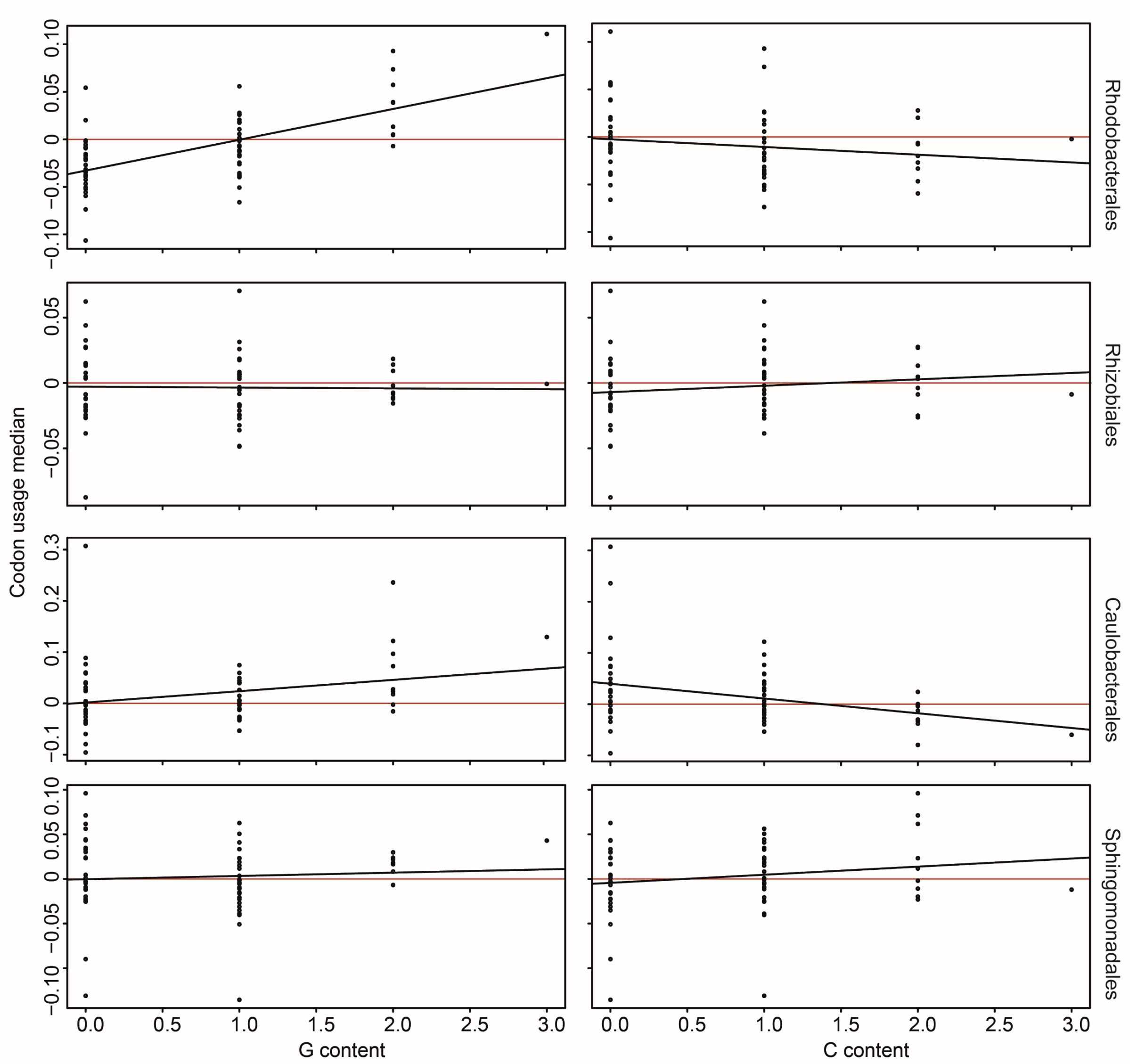

Figure S5. Relationships between codon usage and G or C content. The median relative codon usages for each order were calculated from the mean codon usage values per genome (peak–non-peak/non-peak) and correlated with the G content (left) or C content (right). The trend line is shown in black and line at 0 is in red.

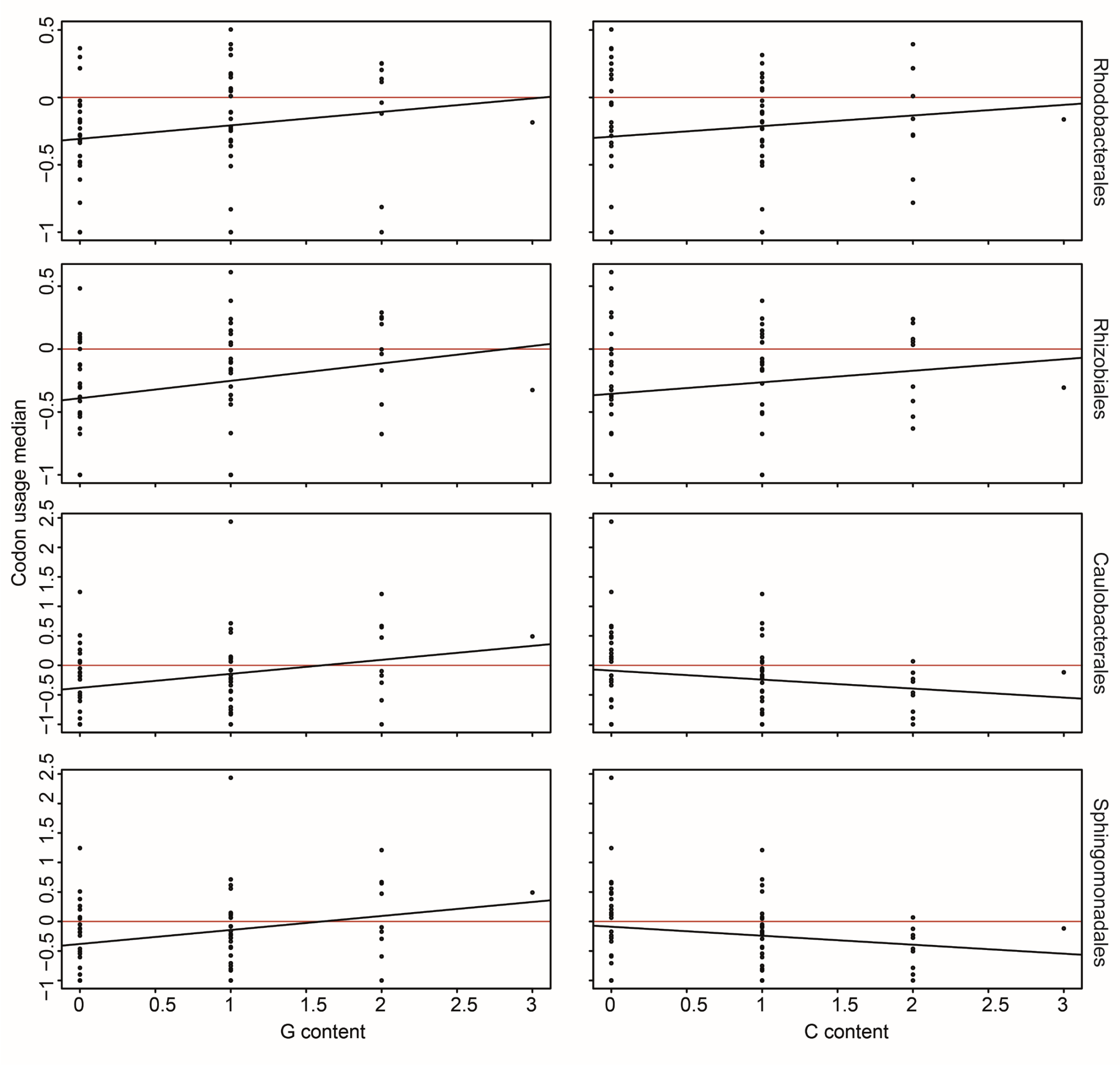

Figure S6. Codon usage of GTA genes vs. non-peak genes relative to the guanine and cytosine content. The median relative codon usages for each order were calculated from the mean codon usage values per genome (GTA/non-peak) and correlated with the G content (left) or C content (right). Homologs of the genes Dshi_2174 and Dshi_2175, encoding the major capsid protein and prohead protease, respectively, were used as representatives for the GTA clusters due to their broad conservation.

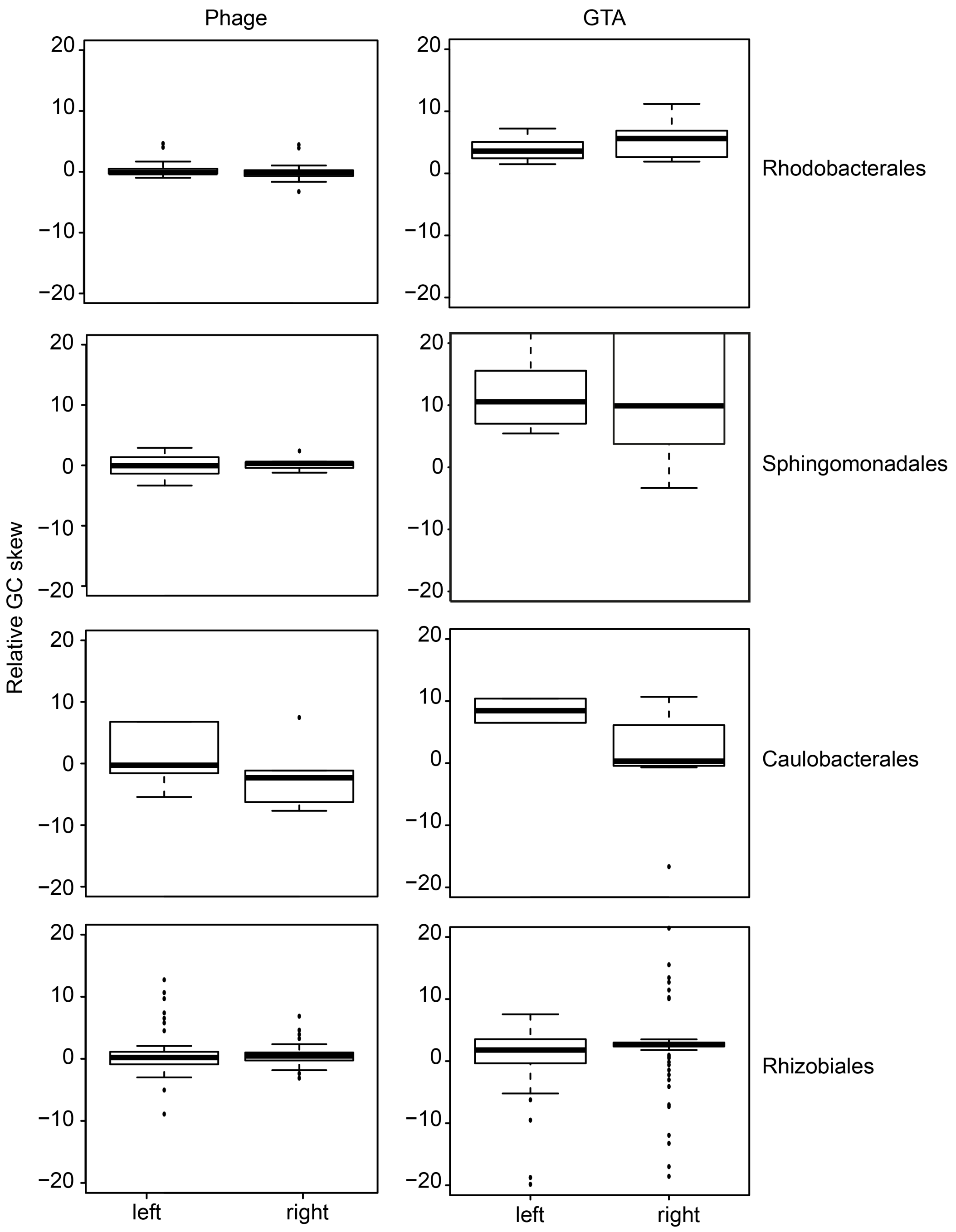

Figure S7. GC skews of phages and GTA gene clusters relative to their respective "host" genomes ((GC skew GTA/Phage-GC skew host)/GC skew host). The genome was divided into the left and right replichores for the analysis, as the values on the right and left replichores would otherwise equalize.
